# A two-stage deep learning framework with progressive feature fusion and attention mechanisms for multi-class retinal disease classification

**DOI:** 10.3389/frai.2026.1925302

**Published:** 2026-09-11

**Authors:** Mithun Vijayan, Goutham Veerapu, Nisha J. S.

**Affiliations:** 1 School of Electronics Engineering, Vellore Institute of Technology, Vellore, India; 2 Computer Science Engineering, Indian Institute of Information Technology, Kottayam, India

**Keywords:** optical coherence tomography, retinal diseases, deep learning, image classification, YOLOv11, attention mechanism, ophthalmology, Grad-CAM

## Abstract

Accurate and efficient automated analysis of Optical Coherence Tomography (OCT) images is critical for large-scale retinal disease screening. However, current deep learning models often fail to simultaneously achieve high classification accuracy, practical computational feasibility, and interpretability. To deal with these problems, this paper presents a deep learning framework on YOLOv11 for eight-class retinal disease classification optimized using a two-stage optimization strategy. The proposed architecture incorporates a Progressive Spatial Fusion (PSF) module to hierarchically integrate multi-scale feature representations, followed by a Squeeze-and-Excitation (SE)-based dual-attention refinement mechanism that refines disease-discriminative features prior to classification. Evaluated on the OCT-C8 dataset, the proposed model achieves 98.00% accuracy, precision, recall, and F1-score, together with 99.70% specificity, while achieving perfect classification performance for the Age-related Macular Degeneration (AMD), Central Serous Retinopathy (CSR), Diabetic Retinopathy (DR), and Macular Hole (MH) classes. Grad-CAM visualizations provide qualitative insights into image regions influencing model predictions and show correspondence with disease-associated structural patterns reported in OCT literature. These results show that the proposed framework provides a good trade-off between classification performance, computational requirements and interpretability and shows potential for automated OCT-based retinal disease screening support.

## 1 Introduction

More than 2.2 billion people globally have a vision impairment or blindness. According to the World Health Organization, nearly one billion of these cases could be prevented or appropriately managed if timely diagnosis and treatment were available (World Health Organization, 2023). The number of patients with retinal diseases is increasing because of the aging of the population, the increasing prevalence of diabetes, and changes in the lifestyle. DR and AMD are the two leading causes of irreversible vision loss among these disorders. These diseases cause progressive and irreversible structural damage. Hence early detection is essential for preserving visual function and improving clinical outcomes.

The need for large-scale retinal screenings has been rising, and therefore there has been an increasing trend toward automation of these systems that could help ophthalmologists, especially in those areas where specialists are scarce. Early diagnosis of retinal disease is crucial for better patient outcomes and minimizing the risk of vision impairment in the long term (He et al., 2023). The evaluation of retinal diseases is enhanced by OCT imaging, which allows for a more precise visualization of retinal morphology (Hassan et al., 2024; Aumann et al., 2019). Artificial intelligence (AI)-assisted OCT analysis has emerged as a promising approach for developing accurate and scalable retinal image analysis systems.

From all retinal imaging technologies, OCT is regarded as the clinical gold standard owing to its superior capability of cross-sectional visualization of retinal structures that cannot be acquired by traditional fundus photography. Unlike fundus imaging, OCT facilitates the visualization of retinal layers in a depth-resolved manner, thus helping to detect subtle changes in the form of intraretinal cysts, drusen deposition, and subretinal fluid accumulation. The use of SD-OCT helps to increase the acquisition rate and spatial resolution as compared to TD-OCT, thus enabling a better visualization of retinal layers as well as changes that are required for the diagnosis of diseases (Han and Jaffe, 2009). These capabilities have established OCT as an effective imaging modality for both retinal disease diagnosis and longitudinal clinical assessment.

The latest development in deep learning has led to remarkable improvements in the automated classification of retinal diseases using OCT images. It has been observed that convolutional neural networks (CNNs) have performed excellently due to their ability to learn hierarchical representations of features from the retina itself, thereby resulting in the reliable detection of different retinal diseases even in cases of variations in image quality and dataset size (Kermany et al., 2018; Fang et al., 2019; De Fauw et al., 2018). More recently, transformer-based and hybrid CNN-transformer architectures have further enhanced representation learning by modeling long-range contextual dependencies, leading to improved performance in several medical imaging tasks. Despite these advances, many existing approaches rely either on single-scale feature extraction or computationally intensive self-attention mechanisms, which may limit their ability to simultaneously preserve fine anatomical details, capture multi-scale contextual information and maintain computational efficiency.

**Figure 1:**
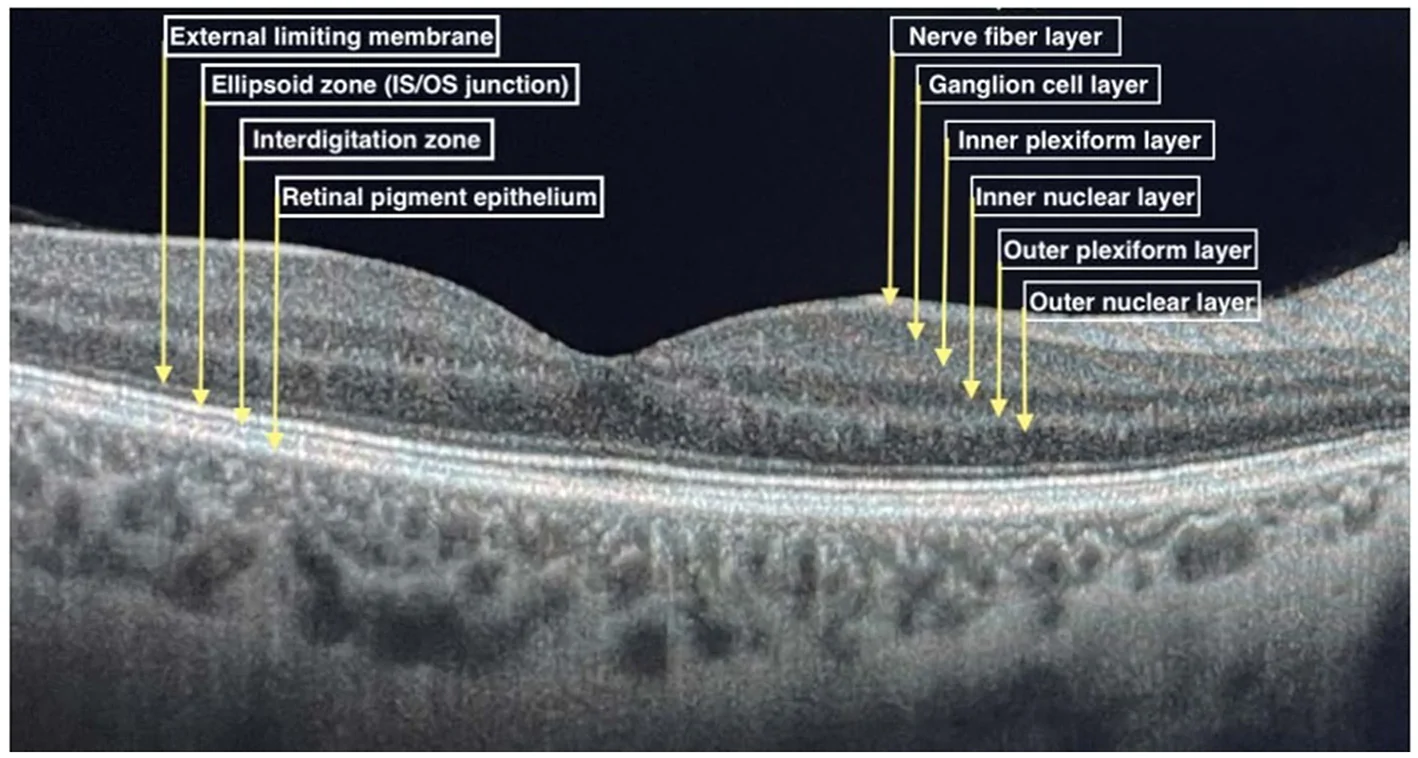
Annotated OCT B-scan of a normal retina highlighting the major retinal layers used as anatomical reference for identifying structural deviations in retinal diseases. Reproduced from Mimier-Janczak et al. (2023) under the CC BY-NC-SA 4.0 license.

**Figure 2:**
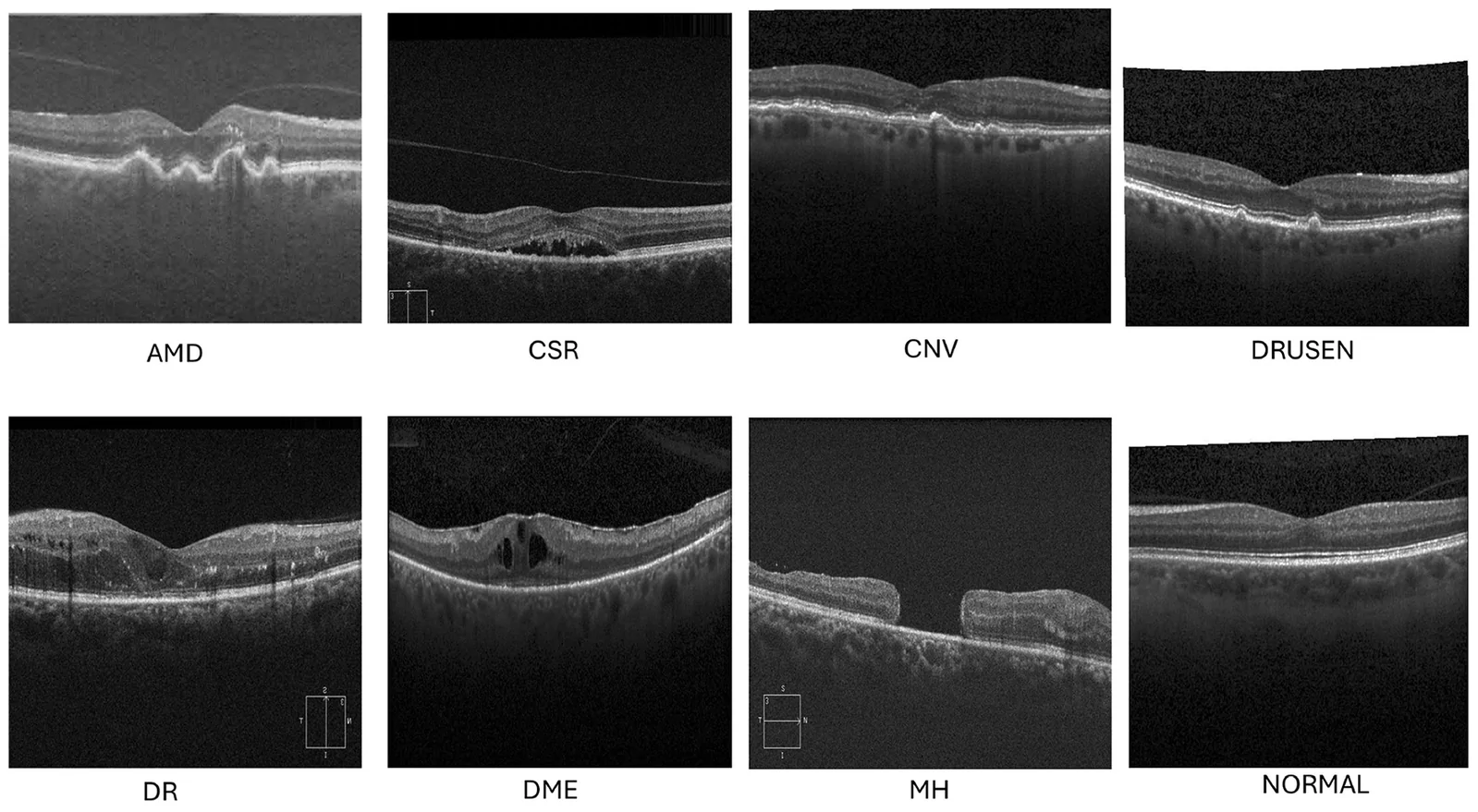
Representative OCT B-scans illustrating different retinal pathologies, alongside a normal OCT B-scan from a healthy eye.

It is common to see the structural manifestations of retinal diseases in terms of minute changes observed in different layers of the retina. For instance, retinal lesions like drusen deposits, intraretinal cysts, subretinal fluid, as well as boundary disruptions in the retina tend to have common features that make it difficult to distinguish among them. Figure 1 illustrates the normal retinal anatomy in a healthy OCT B-scan. Figure 2 presents representative OCT images from the eight retinal disease categories investigated in this study, including drusen, MH, CSR, DME, AMD, CNV, DR, and normal (Akça et al., 2024; Mishra et al., 2022). These examples demonstrate the significant inter-class similarity and intra-class diversity which require feature representations that are able to capture both local and global characteristics.

Motivated by these challenges, this work proposes a YOLOv11-based framework for the classification of eight retinal disease classes. The proposed framework is optimized using a two-stage optimization strategy consisting of classifier adaptation followed by end-to-end fine-tuning. In this regard, the proposed network expands on the YOLOv11-based backbone architecture by including a new Progressive Spatial Fusion (PSF) layer that can hierarchically integrate multi-level features obtained from various layers of the backbone network. The resulting multi-level feature map is then refined using Squeeze-and-Excitation (SE) and spatial attention mechanisms before being processed by a customized classification head for robust disease prediction.

Although attention mechanisms, multi-scale feature fusion strategies, and explainable AI techniques have been widely investigated in medical image analysis, existing OCT classification approaches generally employ these components independently or as isolated enhancement modules. The novelty of the proposed framework lies in the coordinated integration of hierarchical feature fusion and attention-guided refinement within a YOLOv11-based classification architecture. Specifically, the proposed Progressive Spatial Fusion (PSF) module performs progressive alignment and aggregation of intermediate feature representations from different backbone depths, allowing the model to preserve fine retinal structural details while incorporating deeper disease-specific semantic information. Unlike conventional feature fusion methods based on direct concatenation or static aggregation, the proposed PSF strategy enables a structured combination of multi-level representations before attention refinement. Furthermore, the customized classification head adapts the YOLOv11 architecture for OCT-based multi-class retinal disease classification, providing an efficient balance between discriminative capability, computational cost, and interpretability. Therefore, the contribution of this work is not the introduction of individual attention or fusion components, but the design of an OCT-specific framework that coordinates progressive feature fusion, attention-guided refinement, and classification optimization within YOLOv11.

In contrast to standard CNN backbones and CNN-Transformer hybrid models that have been recently developed, the proposed approach provides hierarchical multi-scale feature fusion without resorting to costly computations in terms of transformer modules and high-dimensional representations. Using efficient feature extraction based on convolution and progressive feature fusion and guided by attention mechanisms, the proposed framework achieves an effective balance between enhanced feature representation and computational requirements and model interpretability. These properties are especially desirable in OCT image analysis, since proper recognition of localized abnormalities of retinal structures depends on effective fusion of local structure-specific features and global context information.

The main contributions of this work are summarized as follows:

- A YOLOv11-based classification framework is proposed using a two-stage optimization strategy, where initial classifier adaptation is followed by complete network fine-tuning for improved OCT disease recognition.
- A Progressive Spatial Fusion (PSF) mechanism is introduced to progressively align and integrate intermediate feature representations from different YOLOv11 backbone stages, enabling effective fusion of low-level retinal structural information and high-level disease-specific semantic features.
- A dual-attention refinement mechanism combining channel-wise SE attention and spatial feature refinement is introduced to enhance the fused feature representations and emphasize diagnostically important retinal regions.
- A customized classification head is proposed to convert refined multi-scale representations to reliable predictions for classification of eight classes of retinal diseases, supporting stable optimization and improved generalization.
- Experiments are conducted in terms of statistical analysis, ablation study, interpretability with Grad-CAM, computational efficiency, and external validation to verify the efficiency, robustness, and generalization ability of the proposed framework in automated retinal disease classification.

The remainder of this paper is organized as follows. Section 2 presents the related works and discusses existing approaches for retinal OCT image classification, along with the identified research gaps. Section 3 describes the proposed methodology, including the YOLOv11n baseline classification architecture, the proposed modified YOLOv11+PSF+SE+Head framework with Progressive Spatial Fusion and attention mechanisms, and the adopted two-stage optimization strategy. Section 4 presents the experimental setup, dataset details, preprocessing procedures, evaluation metrics, and implementation configurations. Section 5 discusses the experimental results, including learning dynamics, performance evaluation, statistical analysis, model complexity, and interpretability. Section 6 presents cross-dataset generalization evaluation on an external dataset. Section 7 provides a broader discussion of results and outlines study limitations. Finally, Section 8 concludes the paper and outlines directions for future research.

### 1.1 Overview of dataset pathologies

The OCT-C8 dataset employed in the present research study is comprised of eight different retinal pathological states that are commonly encountered in clinical practice, which include AMD, DME, CNV, DR, CSR, Drusen, MH, and Normal. All the above conditions have different morphological patterns on the OCT images. This enables OCT-based multi-class disease classification. Figure 1 demonstrates an OCT B scan of a normal retina with all the layers well labeled. The clinical description of all the pathologies is given in the Appendix section.

## 2 Related works

In the last few years, OCT image analysis has been found to be highly useful in the automated diagnosis of various retinal diseases. In light of the increased interest in deep learning, many architectures have been developed to achieve higher accuracy and more robust performance. The following section presents an overview of CNN models, attention mechanisms, multi-scale fusion, transformer hybrids, explainability approaches, and multimodal fusion, which provides a foundation for our proposed YOLOv11-based classification system.

### 2.1 CNN-based retinal OCT classification

Initial approaches largely relied on CNNs for feature extraction and classification in retinal imaging. The work of Kermany et al. (2018) provided an early demonstration of deep learning in ophthalmology through the use of InceptionV3 on a four-class dataset. Later works extended this by evaluating models such as VGG, ResNet, and DenseNet on OCT data for retinal disease classification, achieving high diagnostic accuracy. A notable example is the work of Chen et al. (2021) where transfer learning using VGG-19, ResNet-50 and ResNet-101 was performed on an OCT dataset for multi-class AMD and DME with high test accuracies of 99.42%, 99.09%, and 99.19% respectively. Nugroho (2018) also compared DenseNet-169 and ResNet-50 with traditional handcrafted features for four-class classification (CNV, DME, drusen, and normal) and reported accuracies of 88% and 89% significantly outperforming HOG and LBP descriptors. Recently, Sunija et al. (2021) proposed a compact CNN architecture called OctNET for the classification of retinal diseases from OCT images. These networks successfully learned disease-specific features and created a solid benchmark for OCT-based diagnosis. Nevertheless, traditional CNNs suffer from restricted receptive fields and an inability to model cross-layer dependencies which are essential for retinal pathology analysis.

These CNN-based studies provide a strong basis for the analysis of retinal images. However, narrow receptive fields and usually single-scale processing limit their ability to encode fine and coarse retinal structures. This poses a problem for retinal diseases that involve multiple layers and occur at different spatial scales. These limitations directly motivate the multi-scale fusion of our framework.

### 2.2 Attention-enhanced networks

A range of different attention schemes can be used to improve the model's focus on the pathological areas within OCT images. The techniques that are typically used include Squeeze-and-Excitation (SE) blocks, CBAM, BAM, and lesion-aware attention modules. Kugelman et al. (2019) have demonstrated that the integration of SE blocks into OCT segmentation networks allows it to recalibrate channel responses and hence enhances the delineation of complex retinal structures. Fang et al. (2017) proposed a CNN with incorporated spatial attention that underlined the lesion regions for abnormality detection. Nabijiang et al. (2022) introduced the Block Attention Mechanism, integrating channel and spatial attention, and exhibited superior robustness compared to numerous existing methods for OCT classification. Pan et al. (2025) utilized CBAM in a lightweight OCT classifier that improved the localization of disease-specific features. Guo and Zhao (2025) demonstrated that incorporating modules such as ADCCA and ESA into hybrid attention networks can substantially improve feature calibration and overall model performance in OCT image reconstruction tasks.

These models highlight the importance of attention, but most of them show these modules as separate additions instead of putting them together in a hierarchy across different feature scales. This makes it hard for them to use both shallow structural cues and deep semantic signatures at the same time. Our dual attention YOLOv11 framework fixes this by putting SE-based dual-attention refinement mechanism into a progressive multi-scale fusion pipeline that lets layers work together to improve each other.

In recent years, attention-based methods have provided more improvement in classifying OCT images through better feature representations at various spatial scales and contexts. Recently, a hybrid deep learning architecture employing Ant Colony Optimization to optimize its parameters for multi-class OCT classification was suggested by Khan et al. (2023). Attention-guided transfer learning techniques were studied to provide better feature representations for better classification performance (Sivappriya and Kar, 2024). Also reinforcement learning was applied to achieve higher adaptability for multi-class OCT classification (Abbas et al., 2025), and OCTNet was developed by Khalil et al. (2024) as a multi-scale attention fusion network using InceptionV3 that showed promising performance on difficult OCT data. Overall, these studies showed the usefulness of attention mechanisms for better feature representation; however, effective combination of hierarchical multi-scale features with lightweight architectures is still an open problem.

### 2.3 Multi-scale feature fusion approaches

Multiscale feature fusion has played a significant role in improving OCT classification by incorporating information from both fine grained details and the larger context. Rasti et al. (2017) proposed an MCME for classifying macular OCT images. They combined features from different scales to classify a normal retina versus common macular pathologies, including AMD and DME. Das et al. (2021) used a deep CNN to successfully distinguish similar pathologies, such as CNV and drusen, by multiscale fusion. Umer et al. (2023) proposed a deep learning framework that incorporates feature fusion and selection strategies and demonstrated the strength of multiscale feature representation in enhancing the accuracy of OCT based retinal disease classification.

While these approaches give insight into the key role of multiscale integration, most of them depend on static concatenation or parallel fusion branches. In contrast, our proposed Progressive Spatial Fusion (PSF) module performs dynamic hierarchical merging of intermediate YOLO feature maps, allowing more effective discrimination of subtle retinal abnormalities.

### 2.4 Transformer-based and hybrid architectures

Hybrid models, which couple CNNs with transformers, have lately become prevalent in OCT analysis. Wang et al. (2023) presented OCTformer, an efficient hierarchical transformer network adapted for retinal OCT image recognition, effectively integrating both the local and global contextual features. Similarly, Laouarem et al. (2024) proposed HTC Retina, a CNN-Transformer hybrid for retinal classification, leveraging the complementary strengths of convolutional and attention mechanisms. Transformer-based architectures have been further advanced in OCT analysis by a multiscale CNN Swin Transformer hybrid with boundary supervision. Fan et al. (2025) applied such a model to multi-class biomarker segmentation and OCT image classification. They illustrated high accuracy and excellent generalization performance. However, it is difficult to use transformer-based models given their high computational complexity and typically huge amounts of data needed to converge. The proposed work presents the YOLOv11 backbone that provides an efficient alternative to transformer-based models without the loss of fine spatial details and contextual awareness with a convolution-based design that avoids the additional self-attention computation used in transformer architectures.

Compared with these transformer-based designs, which often demand large datasets and incur high computational cost, the YOLOv11 backbone provides a computationally feasible alternative that maintains rich spatial detail and long-range context with reduced architectural complexity.

### 2.5 Explainable AI (XAI) in retinal OCT analysis

Recently, explainable AI has gained high relevance for the interpretation of deep learning model predictions in medical imaging. For instance, Grad-CAM emphasizes the most influential regions contributing to model decisions, hence allowing for interpretable analysis of deep networks. Further refinements of the visualization of salient regions are given by the related techniques XGrad-CAM and SmoothGrad-CAM++. The concordance between generated attention maps and disease-relevant structures was found to be high in several studies applying these methods in retinal OCT analysis. Grad-CAM has also been integrated into guided attention strategies, where generated heatmaps supervise attention modules to enforce more pathology aware learning (Duvvuri et al., 2022; Raveenthini et al., 2025; Jaimes et al., 2025). Here, visualizations from Grad-CAM are used to interpret the YOLOv11+SE+PSF+Head model's decision regions, with a view toward supporting transparency and interpretation of model predictions.

### 2.6 Multimodal integration and diagnostic fusion

While XAI techniques improve trust in AI-driven OCT analysis, other research directions have investigated the expansion of diagnostic capability by using a multimodal integration approach. Beyond single-modality CNNs, integrated systems that incorporate OCT with modalities such as fundus photography or MRI have also shown great promise. Zhang et al. (2024) employed a method of multimodal deep transfer learning that combined OCTA features with clinical data to predict the recurrence of macular edema after anti-VEGF therapy with high accuracy. Jin et al. (2022) developed a deep learning framework that was integrated with OCT and OCTA images to classify the activity of choroidal neovascularization in AMD. Their approach produced 95.5% accuracy (AUC 0.9796) on both internal and external datasets. These developments of multimodal fusion, ensemble methods, proper data augmentation techniques, and efficient networks, have further enhanced the model generalizability and applicability in the real world.

Recent advances in ocular disease analysis have also explored multi-disease classification using fundus imaging. Fundus-DeepNet introduced a multi-label deep learning framework that utilizes feature fusion from fundus images along with attention-based feature refinement to improve the detection of multiple ocular diseases (Al-Fahdawi et al., 2024). Similarly, Alqassab et al. (2026) proposed a hybrid quantum convolutional neural network framework that integrates classical convolutional feature extraction with quantum-based representation learning for multi-disease ocular classification from fundus images. These studies demonstrate the effectiveness of advanced feature learning and fusion strategies for automated ocular disease diagnosis. However, fundus images mainly provide two-dimensional surface-level retinal information, whereas OCT imaging enables depth-resolved visualization of retinal layers. Therefore, OCT-based classification requires specialized models capable of capturing subtle layer-specific abnormalities, multi-scale structural variations, and complex pathological patterns.

### 2.7 Summary of research gaps

Recent advances in CNN-based, attention-guided, multi-scale fusion, and transformer-based models have substantially improved OCT image classification. Nevertheless, no single approach adequately addresses the diverse structural characteristics of retinal pathologies. Conventional CNNs primarily learn features at a single scale, making it difficult to represent abnormalities distributed across multiple retinal layers. Although attention mechanisms improve the representation of diagnostically important regions, they often model either channel or spatial information independently and therefore provide limited interaction across feature scales. Likewise, many feature fusion networks rely on fixed aggregation strategies that are unable to fully exploit the complementary information contained in shallow and deep representations. Transformer-based methods enhance the global context modeling but generally require higher computational resources and bigger training datasets, limiting their applicability in real-time clinical environments. These limitations collectively delineate a clear void in which existing approaches do not simultaneously deliver joint multiscale discrimination, computational feasibility, and interpretability in a single architecture. Therefore, the remaining challenge is not the independent use of attention or feature fusion mechanisms but the development of an efficient framework that can jointly exploit hierarchical multi-scale representations, adaptive feature refinement, and classification capability for OCT disease recognition. Table 1 summarizes these limitations in existing studies and details how the proposed YOLOv11+PSF+SE+Head framework addresses them. In view of the above observations, the proposed YOLOv11+PSF+SE+Head network consists of three important building blocks:

- Progressive Spatial Fusion (PSF) for hierarchical fusion of multi-scale feature representations.
- Dual attention techniques for concurrent improvement of channel-wise and spatial feature maps.
- Grad-CAM based interpretability analysis to provide qualitative insights into the regions influencing model predictions.

**Table 1:** Summary of limitations in existing OCT-based retinal disease classification studies and how the proposed YOLOv11+PSF+SE+Head framework addresses them.

Category	Representative studies	Limitations of existing methods	Proposed solution
CNN-based OCT classification	(Kermany et al., 2018; Chen et al., 2021; Nugroho, 2018; Sunija et al., 2021)	Rely primarily on single-resolution feature representations and limited hierarchical feature interaction, which may restrict the modeling of complex multi-layer retinal structures.	Employs progressive multi-scale spatial fusion to dynamically integrate shallow structural cues with deep semantic representations.
Attention-enhanced networks	(Kugelman et al., 2019; Nabijiang et al., 2022; Guo and Zhao, 2025)	Most attention-based OCT models incorporate channel or spatial attention at specific stages, but lack a unified progressive multi-scale fusion strategy across hierarchical backbone levels.	Integrates dual attention (SE and spatial attention) within the progressive fusion hierarchy to enable cross-scale feature refinement.
Multiscale fusion approaches	(Rasti et al., 2017; Das et al., 2021; Umer et al., 2023)	Static concatenation or parallel fusion strategies with weak interaction between shallow and deep features.	Introduces Progressive Spatial Fusion (PSF) for hierarchical and dynamic merging of intermediate YOLO feature maps.
Transformer-based and hybrid models	(Wang et al., 2023; Laouarem et al., 2024; Fan et al., 2025)	Transformer-based OCT models typically incur higher computational overhead and data requirements than conventional CNNs, motivating hybrid designs and efficiency-oriented modifications.	Utilizes a YOLOv11n backbone combined with progressive feature fusion and attention refinement to achieve improved feature representation with practical computational requirements.
Explainable AI in OCT analysis	(Raveenthini et al., 2025; Jaimes et al., 2025)	Explainability techniques primarily used as post-hoc analysis without architectural validation.	Employs Grad-CAM to analyze decision regions and assess attention consistency, supporting interpretability and clinical trust.

The following section describes the proposed framework, including its feature fusion strategy, attention guided architecture, and two-stage optimization procedure.

## 3 Methodology

### 3.1 YOLOv11-based classification model

The proposed framework uses the classification architecture of YOLOv11 as the backbone due to its effective feature extraction capability and compact backbone design. Earlier YOLO versions were mainly for object detection, but YOLOv11 has a classification architecture dedicated to and optimized for image-level recognition, while still keeping the compact design and hierarchical feature learning of the original network (Jocher and Qiu, 2024). This makes it well suited for retinal OCT image classification, as both computational efficiency and discriminative feature representation are important.

The YOLOv11 classification architecture consists of two main components:

- Backbone—Extracts hierarchical feature representations from the input OCT image.
- Head—Produces the final probability distribution over the retinal disease categories.

#### 3.1.1 Backbone: hierarchical feature extraction

The backbone progressively extracts image features through multiple convolutional stages operating at different spatial resolutions. It comprises standard convolutional layers together with the C3K2 and C2PSA blocks, which improve feature representation while maintaining a compact backbone design.

- Conv blocks: A standard Conv Block is a core component that contains a Conv2D layer (with a 3 × 3 kernel), a batch normalization layer, and a non-linear activation such as ReLU or SiLU. A few convolution blocks reduce the spatial dimensions from H × W to H/2 × W/2 by downsampling with stride 2.
- C3K2 blocks: YOLOv11 uses the C3K2 module instead of the standard Cross Stage Partial (CSP) bottleneck to improve the feature extraction with fewer parameters. The module executes the transformation of the input through parallel transformation and shortcut branches, followed by the aggregation of their outputs, enabling efficient information propagation and finer structural details preservation. C3K2 provides a better trade-off between feature representation ability and computational cost compared to previous C2f-based designs.
- C2PSA blocks: C2PSA enhances the feature extraction by embedding Position Sensitive Attention (PSA) into the Cross-Stage Partial network. Then spatial attention is used to attend to those regions of the retina that contain more information. At the same time, the network keeps the broader context shared among the feature maps. Due to this selective feature refinement, the network can pick up on very tiny pathological details that are crucial for retinal OCT classification and, at the same time, it doesn't get too heavy on computation.

#### 3.1.2 Head: adaptive classification module

Unlike the variants of YOLO intended for detection, the classification architecture does not have a separate neck module but directly links the backbone to a compact classification head. Initially, the final feature maps are transformed into a compact representation by the Global Average Pooling (GAP) method that reduces the number of trainable parameters while keeping the most discriminative information. Next, the pooled feature vector goes through a series of fully connected layers, and its output is subjected to a Softmax activation that gives the probability for each category of retinal disease.

#### 3.1.3 Advantages of YOLOv11 for classification

YOLOv11 has several benefits when compared to conventional CNNs and transformer-based architectures in image classification tasks. Its architecture can be optimized by removing the detection-specific layers and adding a compact classification head that makes it optimized for efficient inference. C3K2 blocks further enhance feature extraction by capturing rich spatial details with fewer parameters. Furthermore, the C2PSA attention mechanism incorporated within the YOLOv11 backbone contributes to enhanced feature representation by capturing fine-grained spatial information, providing a suitable foundation for retinal disease classification. Due to its efficient backbone design, YOLOv11 provides a suitable foundation for real-time OCT image analysis. However, the proposed PSF and attention modules introduce additional computational operations to improve multi-scale feature representation and disease-specific feature refinement. Compared with widely used classification backbones such as ResNet, DenseNet, EfficientNet, and ConvNeXt, the YOLOv11 classification architecture provides a favorable foundation for OCT image classification through its efficient C3K2-based hierarchical feature extraction, optimized backbone design, and computationally efficient feature representation capability. Although transformer-based models such as Swin Transformer and Vision Transformer (ViT) provide strong global context modeling, their higher computational requirements and dependency on large-scale training data can limit their practical deployment in resource-constrained clinical environments. Therefore, YOLOv11 was selected as the backbone of this work because its compact convolutional design, efficient feature extraction blocks, and classification-optimized architecture provide an appropriate foundation for OCT image classification while maintaining accuracy and efficiency. Building upon these advantages, the proposed framework further enhances YOLOv11 through Progressive Spatial Fusion, dual attention-guided refinement, and a customized classification head for improved multi-scale retinal disease representation.

#### 3.1.4 Selection of the YOLOv11 variant

**Figure 3:**
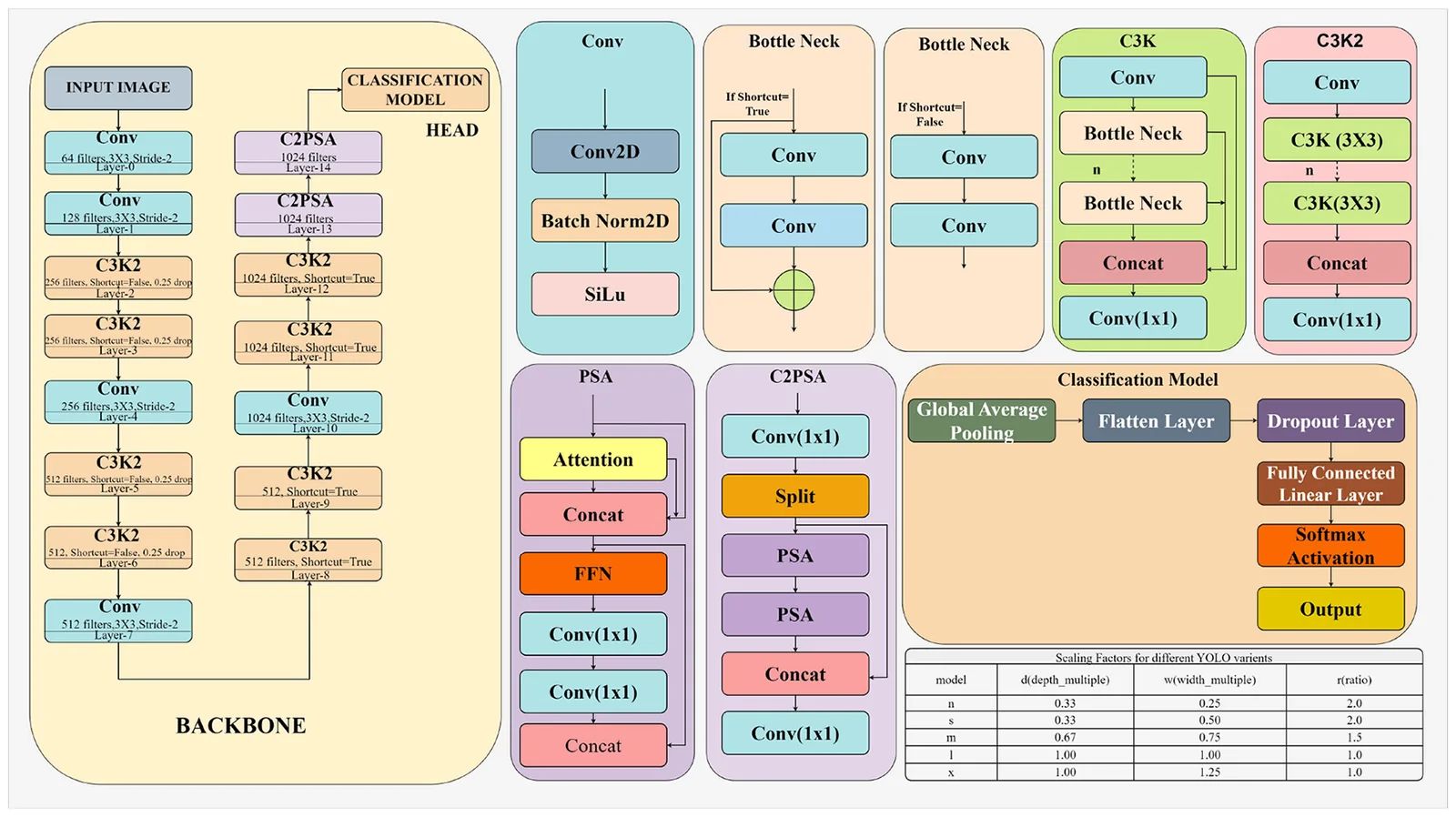
YOLOv11 architecture. The backbone consists of multiple Conv and C3K2 blocks, while the head contains the classification module.

YOLOv11 has five variants: nano (YOLOv11n), small (YOLOv11s), medium (YOLOv11m), large (YOLOv11l), and extra large (YOLOv11x). They all have different trade-offs with computational complexity and prediction performance. The nano variant was selected from these because of its optimal trade-off between model size, inference speed and classification accuracy for real-time retinal OCT analysis. Figure 3 illustrates the overall architecture of the adopted YOLOv11n classification model.

### 3.2 Proposed modified YOLOv11n+PSF+SE+Head model

**Figure 4:**
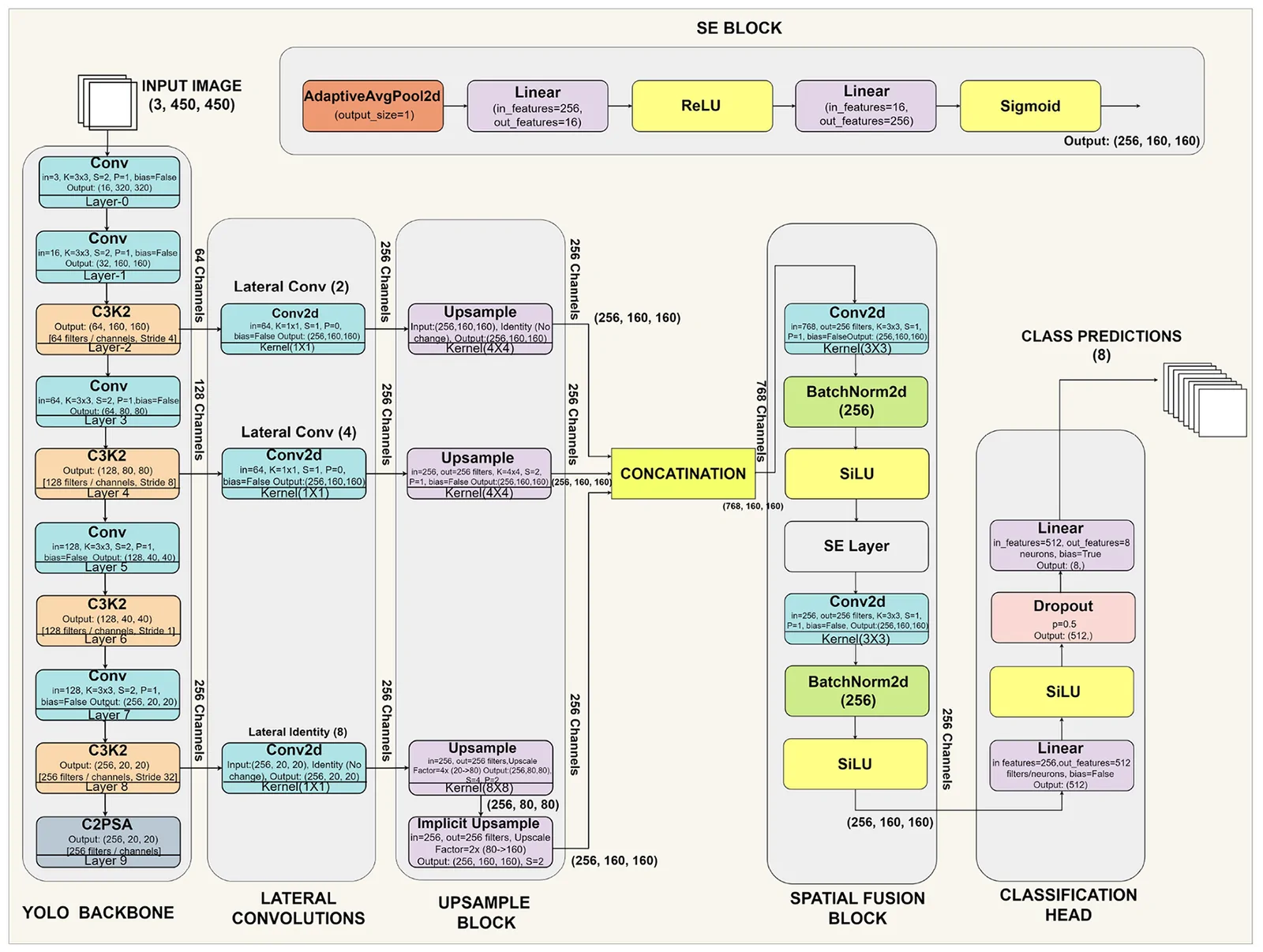
Architecture of the proposed YOLOv11-based classifier. The model integrates the YOLO backbone with lateral convolutions, Progressive Spatial Fusion, attention-guided feature refinement, and a customized classification head. The fusion block incorporates SE-based channel recalibration and spatial feature refinement to enhance disease-specific representation learning.

The full end-to-end architecture and processing pipeline of the proposed YOLOv11+PSF+SE+Head framework is illustrated in Figure 4, which demonstrates the transformation of an input OCT image into the final prediction of retinal disease through the hierarchical feature extraction, Progressive Spatial Fusion (PSF), attention-guided refinement, and the modified classification head. Then, the proposed Progressive Spatial Fusion (PSF) module inputs these intermediate feature maps and achieves channel alignment, learnable upsampling and progressive multi-scale fusion to obtain a unified feature representation. The fused features are subsequently refined using Squeeze-and-Excitation (SE) and spatial attention mechanisms to emphasize disease-discriminative retinal structures. Finally, the refined feature representation is passed through the modified classification head to predict one of the eight retinal disease classes. The complete network is optimized using a two-stage training strategy consisting of classifier adaptation followed by end-to-end fine-tuning. This proposed system is built upon the basic YOLOv11n classification network by adding a Progressive Spatial Fusion (PSF) module, dual attention mechanisms, and a new classification head design. These features are aimed at enhancing multi-scale feature representation while maintaining practical computational feasibility for OCT image classification. The proposed architecture combines hierarchical feature fusion and attention-guided refinement to capture the local structural details and high-level semantic information existing in OCT images.

#### 3.2.1 Progressive spatial fusion module

The proposed PSF module is designed to aggregate complementary information from different stages of the YOLOv11 backbone before classification. Unlike conventional feature fusion methods that rely on direct concatenation or top-down pathways, the proposed module progressively aligns and integrates feature maps obtained at different network depths, thereby preserving both spatial resolution and semantic information.

The PSF module integrates features from three key layers of the backbone: layer 2 (high resolution, early features, 64 channels), layer 4 (medium resolution, mid-level features, 128 channels), and layer 8 (low resolution, high-level semantic features, 256 channels). Together, they provide complementary information required for accurate characterization of retinal morphology.

Feature fusion is performed in three sequential stages.

**Channel alignment:** A 1 × 1 convolution is applied to align the channel dimensions of feature maps in layer 2 and 4 to the target of 256 channels, just like layer 8. Layer 8 already has the same number of channels as the target, so it passes through directly without modification.

**Learnable upsampling:** Feature maps from lower resolutions (layers 4 and 8) are upsampled to the size of the highest resolution feature map (originally from layer 2). Instead of using simple interpolation techniques, transposed convolutions, also known as deconvolutional layers are used for upsampling. This enables the model to learn how to choose optimal deconvolution filters which preserve important spatial information during upsampling, thus allowing for more accurate feature alignment. Specifically:

- Layer 4 features (128 channels, stride 8) are upsampled using a transposed convolution with a kernel size of 4, stride of 2, and padding of 1 to match layer 2's resolution (stride 4).
- Layer 8 features (256 channels, stride 16) are upsampled using a transposed convolution with a kernel size of 8, stride of 4, and padding of 2 to match layer 2's resolution (stride 4).

**Progressive feature fusion:** The feature maps from Layers 2, 4, and 8 are combined by concatenation along the channel dimension in a process that matches both their channels and resolutions. This generates a unified feature representation that merges high-resolution spatial details from earlier layers with the deeper semantic context of later layers at multiple scales. The common spatial resolution is maintained in the concatenated feature map, as in Layer 2, and channel depth is expanded, hence providing a full feature set combining fine details and global context needed by the model for accurate classification.

**Figure 5:**
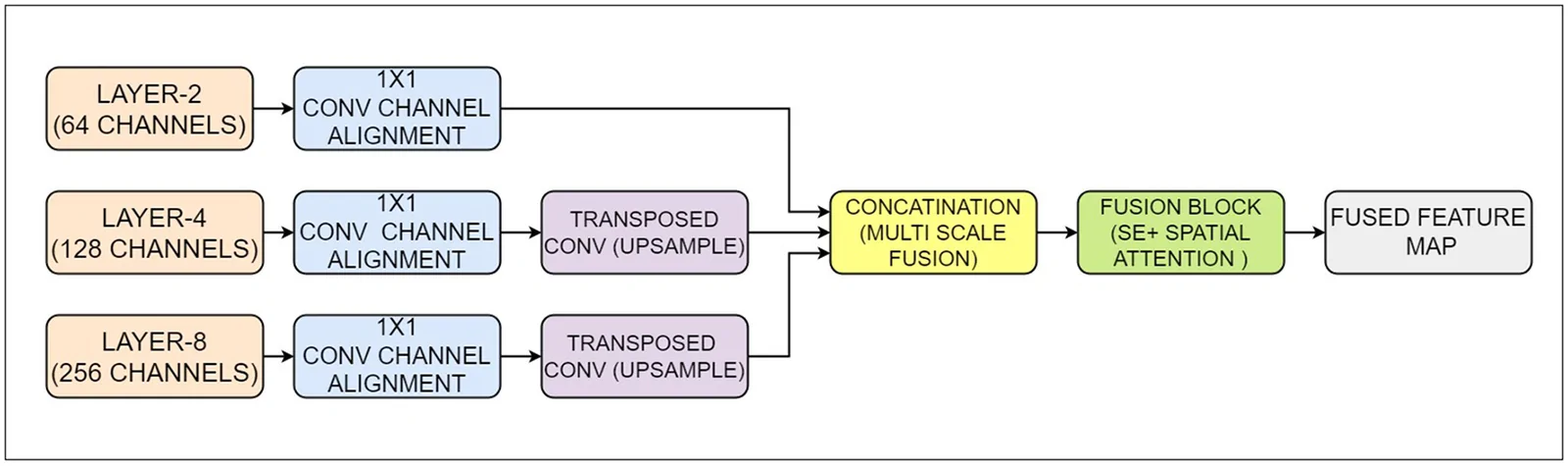
Internal architecture of the Progressive Spatial Fusion (PSF) module showing multi-scale feature integration, dual-attention refinement, and generation of the fused feature map.

The internal workflow of the proposed Progressive Spatial Fusion (PSF) module is shown in Figure 5. After gradually aligning and upsampling intermediate feature maps from various backbone layers, it concatenates them into a single multi-scale representation and uses dual attention (SE and spatial) to refine the fused output before sending it to the classification head. In contrast to conventional top-down fusion techniques like FPN (Lin et al., 2016) or PANet (Liu et al., 2018), the suggested PSF module preserves both semantic depth and spatial precision by performing progressive alignment and upsampling across backbone layers. This design can better capture the retina's fine structures and pathological patterns, which will help improve the accuracy of diagnoses.

#### 3.2.2 Dual attention-based feature refinement

The resulting multi-scale feature map, after spatial concatenation, is fed into a fusion block. The fusion block incorporates an SE-based dual-attention refinement mechanism consisting of channel-wise SE recalibration and spatial feature refinement. In the proposed framework, spatial attention is incorporated as part of the integrated dual-attention refinement process rather than being introduced as a separate standalone module. The objective of this design is to jointly exploit channel-wise feature recalibration through SE attention and spatial feature refinement for improved disease-specific representation learning. These modules refine feature representation on both channels and spatial locations, by means of which the model is allowed to enhance its focus on features critical for correct diagnosis.

The SE channel attention component forms the beginning of the fusion block. This mechanism models dependencies between channels through active feature response adaptation of each channel. First, the global average pooling across the spatial dimensions produces a channel descriptor. This descriptor passes through two fully connected layers with ReLU and sigmoid activations to output the final channel-wise weights. These weights multiply with the original feature map on a channel-by-channel basis. Such a mechanism thereby allows the model to enhance the key channels while suppressing the lesser informative ones, hence improving the feature selectivity for classification.

The channel refined feature map is subsequently processed through convolutional layers that progressively integrate spatial attention. The purpose of such layers is learning to attend to various regions within the feature map, enabling the model to attend to the most significant regions of the feature map. This further enhances the representation by focusing on spatially relevant regions while suppressing irrelevant background information. This ensures that all the spatial locations associated with pathological regions receive due attention. By operating in combination, channel-based SE attention and implicit spatial attention allow the model to effectively extract the most useful features, both from a channel perspective and a spatial one.

#### 3.2.3 Modified classification head

The classification head is redesigned to adapt the YOLOv11 classification architecture for retinal OCT multi-class classification. Unlike the standard detection head that predicts bounding boxes and object classes, the proposed design replaces the detection-specific components with layers dedicated to image-level classification of eight retinal disease categories. The refined feature map generated by the spatial fusion block is first processed using a Global Average Pooling (GAP) layer, which converts the spatial feature maps into a fixed-length feature vector while retaining the most discriminative information and reducing sensitivity to spatial variations. GAP minimizes the number of trainable parameters, but it also ensures simpler feature representation and mitigates the risk of overfitting in case of limited training data. The resulting feature vector is subsequently passed through a series of fully connected layers that progressively learn higher-level feature representations for retinal disease classification. Each linear layer is followed by a Sigmoid Linear Unit (SiLU) activation function to capture delicate pathological changes, and Dropout layers are appended to improve the generalization capability. The feature vector output is then fed into the dense layer, which generates logits of eight classes of retinal diseases. The softmax function outputs a probability distribution over the eight classes and the class with the maximum probability is the final prediction. In contrast to the original classification head of YOLOv11 architecture, the proposed architecture permits deeper feature transformations and retains a compact architecture, enabling better differentiation of subtle retinal pathologies in automated OCT image classification. The integration of hierarchical feature extraction, progressive feature fusion, attention-guided refinement, and two-stage optimization enables the proposed framework to effectively capture both local retinal abnormalities and global structural context for robust retinal disease classification.

## 4 Experimental setup

### 4.1 Dataset description

This proposed methodology was tested using the publicly available OCT-C8 dataset (version 2021) (Naren, 2021) which consists of 24,000 retinal OCT scans divided into eight classes, which include AMD, DR, CSR, DME, CNV, Drusen, MH, and Normal. These datasets have been provided in JPEG image format and consist of 18,400 training samples, 2,800 validation samples, and 2,800 testing samples. Each subset directory is again organized into specific subfolders for each class. Statistics for these datasets are shown in Table 2.

**Table 2:** Class distribution of the retinal OCT dataset used for training, validation, and testing.

Class	Description	Train	Val.	Test	Total
AMD	Age-related macular degeneration	2,300	350	350	3,000
CNV	Choroidal neovascularization	2,300	350	350	3,000
CSR	Central serous retinopathy	2,300	350	350	3,000
DME	Diabetic macular edema	2,300	350	350	3,000
DR	Diabetic retinopathy	2,300	350	350	3,000
DRUSEN	Yellow deposits under the retina	2,300	350	350	3,000
MH	Macular hole	2,300	350	350	3,000
NORMAL	Healthy eyes with no abnormalities	2,300	350	350	3,000
**Total**	**Aggregate dataset size**	**18,400**	**2,800**	**2,800**	**24,000**

The OCT-C8 dataset provides predefined training, validation, and testing partitions organized at the image-level. Since patient identifiers or subject-level metadata are not available in the publicly released dataset, patient-level separation cannot be assessed from the available information. Therefore, the experiments in this study were conducted using the official dataset partitions provided by the dataset authors to ensure consistency and reproducibility of the benchmark evaluation protocol.

### 4.2 Data preprocessing and augmentation

To ensure uniform input dimensions and enhance generalization, all images were preprocessed using a series of steps. All OCT images were resized to 450 × 450 pixels using zero padding when necessary to preserve the original aspect ratio. The OCT images were loaded as three-channel RGB inputs using OpenCV to satisfy the input requirements of the pretrained YOLOv11 classification backbone. Since the proposed framework utilizes ImageNet-pre-trained weights, ImageNet normalization was applied to maintain compatibility with the learned feature representations of the pre-trained model. During the training phase, data augmentation was used to improve robustness against variations in OCT image appearance. These augmentations included random horizontal flipping, brightness and contrast adjustment, rotation, shifting, random zooming, and blurring. Data augmentation was applied during training using the Albumentations library. The complete augmentation settings are summarized in Table 3.

**Table 3:** Data augmentation methods with their parameter configurations for model training.

Augmentation method	Configuration
Horizontal flip	Probability = 0.5
Random contrast and brightness	Contrast limit = 0.2; Brightness limit = 0.2
Rotation	Limit = ±10°
Scale, shift, rotate	Scale limit = 0.1; Shift limit = 0.1; Rotate limit = 10°
Gaussian blur	Kernel size = (3, 3); Probability = 0.1
Random zoom	Zoom range = [0.9, 1.1]

### 4.3 Hardware and training setup

All the experiments have been performed using Google Colab Pro with a Tesla T4 GPU having 15,360 MiB of VRAM. Implementation has been done using Python 3.11 version along with PyTorch 2.5.1 and CUDA 12.4. To improve computational efficiency as well as decrease the use of VRAM, training was done using PyTorch Automatic Mixed Precision (AMP). All the experiments related to training, validation, and testing have been conducted within the same runtime environment for consistency.

### 4.4 Hyperparameters

The optimizer, learning rate schedule, and training hyperparameters were selected to suit the proposed YOLOv11+PSF+SE+Head framework. The complete set of hyperparameters used in all experiments is listed in Table 4.

**Table 4:** Hyperparameter settings.

Hyperparameter	Value
Model backbone	YOLOv11n
Total training epochs	40 (10 head-only + 30 full fine-tuning)
Batch size	16
Initial learning rate	8.33 × 10^−4^ (auto-tuned by Ultralytics)
Optimizer	AdamW
Learning rate scheduler	Cosine annealing with warm restarts
Weight decay	Default (Ultralytics configuration)
Loss function	CrossEntropyLoss
Mixed precision	Enabled (torch.cuda.amp.autocast)
Evaluation frequency	After each epoch
Augmentation library	Albumentations (with ImageNet normalization)
Model evaluation device	GPU (Tesla T4)

### 4.5 Training strategy and model evaluation

In order to achieve improved training effectiveness and generalization capabilities, a two-stage optimization strategy was adopted. The proposed YOLOv11-based architecture was optimized through two sequential training phases while maintaining a fixed network architecture throughout the training process. The first phase focused on classifier head adaptation by training the newly introduced classification layers while preserving the pre-trained backbone representations. Subsequently, the second phase performed end-to-end fine-tuning of the complete network to refine feature representations and improve disease-specific classification performance.

For the initial phase (Epochs 1 to 10), training was restricted to the classifier alone, keeping the layers of the YOLOv11 backbone frozen to ensure the retention of pre-trained weights. Such an approach facilitates rapid adaptation of the model to the target OCT dataset and helps avoid catastrophic forgetting, which generally takes place during fine-tuning of all layers right from the beginning. By limiting updates to the classifier, the model focused on learning dataset-specific decision boundaries efficiently.

For Stage two (epochs 11–40), all layers of the neural network were unlocked and subjected to training in a feed forward process. Full fine-tuning at this stage aids the model to refine its deep features. This is important for the effective discrimination of the small pathological features in the retinal OCT images. In this stage, the learning rate was slowly decreased using a cosine annealing method. This was for stability of convergence and avoiding overshooting.

In both the phases, the mixed precision training technique was applied using the PyTorch AMP module. It greatly boosted the efficiency of the process through faster training and reduced memory usage without jeopardizing the accuracy of the model. The Cross Entropy Loss (CEL) (Goodfellow et al., 2016; Zhang and Sabuncu, 2018) function was used for optimization since it is good for balanced multiclass classification tasks. The CEL evaluates the dissimilarity between the predicted probabilities and the corresponding true class labels. For a problem with *N* classes and a batch size of *M*, the Cross Entropy Loss (CEL) function is defined in Equation 1 as follows:


$$\begin{array}{l} L_{CE}=-\frac{1}{M}\overset{M}{\underset{i=1}{\sum }}\overset{N}{\underset{c=1}{\sum }}y_{i,c}\log\left(p_{i,c}\right) \end{array}$$ (1)

where *y*_*i,c*_ denotes the ground truth label (1 if sample *i* belongs to class *c*, otherwise 0), and *p*_*i,c*_ represents the predicted probability that sample *i* belongs to class *c*. CEL is well suited for this task, as its stable gradients and reliable optimization behavior make it effective for mutually exclusive multiclass problems such as the eight-class retinal OCT classification.

Validation and test evaluations were carried out following each epoch, enabling ongoing monitoring of the model's performance and early detection of any overfitting.

Together with effective mixed precision training and a robust evaluation pipeline, this two-stage approach enabled the model to achieve strong performance and generalization on the retinal OCT classification task.

### 4.6 Performance evaluation metrics

To thoroughly assess the effectiveness of the proposed YOLOv11-based deep learning method for multiple-class classification of retinal OCT images, several evaluation metrics were employed. The combination of these metrics provides a characterization of the prediction power of the proposed model. Much of this is about how to deal with clinically meaningful errors. These errors are often due to false positives and false negatives in medical imaging.

The confusion matrix (Sammut and Webb, 2011) forms the basis of this analysis, which provides the relation between the predicted value and the actual value. Each cell in the confusion matrix belongs to one of the four types: True Positive (TP), meaning the instances which are correctly predicted as positive instances; True Negative (TN), meaning the instances which are correctly predicted as negative instances; False Positive (FP) (also called Type I error), meaning the instances which are incorrectly predicted as positive instances; False Negative (FN) (Type II error), meaning the instances which have been missed out. In this representation, *C*_*ij*_ represents the element of the confusion matrix for instances of class *i* being predicted as class *j*, where *N* represents the number of classes. In order to avoid any confusion while deriving the formulae, the four cell types are represented by separate symbols. For each class *i*, the instances which are correctly identified are *TP*_*i*_, instances which are missed are *FN*_*i*_, instances which are incorrectly classified are *FP*_*i*_, and instances which are correctly rejected are *TN*_*i*_. These quantities are formally defined in Equations 2–5 as follows:


$$\begin{array}{l} TP_{i}=C_{ii}, \end{array}$$ (2)

$$\begin{array}{l} FN_{i}=\overset{N}{\underset{j=1}{\sum }}C_{ij}-TP_{i}, \end{array}$$ (3)

$$\begin{array}{l} FP_{i}=\overset{N}{\underset{j=1}{\sum }}C_{ji}-TP_{i}, \end{array}$$ (4)

$$\begin{array}{l} TN_{i}=\overset{N}{\underset{j=1}{\sum }}\overset{N}{\underset{k=1}{\sum }}C_{jk}-\left(TP_{i}+FP_{i}+FN_{i}\right), \end{array}$$ (5)

#### 4.6.1 Accuracy

Accuracy is considered the main performance metric, as it measures the validity of the predictions made by the model. This measure is equal to the ratio of correct predictions to the total number of predictions. The value of accuracy being high usually indicates that the classifier is able to predict most of the cases correctly. This measure can be misleading when there is class imbalance in the dataset. The class-wise accuracy metric is defined in Equation 6 as follows:


$$\begin{array}{l} {\text{Accuracy}}_{i}=\frac{TP_{i}+TN_{i}}{TP_{i}+FP_{i}+FN_{i}+TN_{i}} \end{array}$$ (6)

#### 4.6.2 Sensitivity (recall)

The sensitivity (or recall) shows how many of the true positives the model can correctly identify. The sensitivity should be high in the case of medical images because failing to detect the disease might result in grave consequences. High sensitivity means that the model accurately detects the positive instances. The sensitivity metric is defined in Equation 7 as follows:


$$\begin{array}{l} {\text{Sensitivity}}_{i}=\frac{TP_{i}}{TP_{i}+FN_{i}} \end{array}$$ (7)

#### 4.6.3 Specificity

The specificity which is also known as the true negative rate is the proportion of true negatives that are accurately predicted by the algorithm. High specificity is extremely crucial for clinical situations because the algorithms can thereby reduce the number of false positives and avoid classifying healthy patients as diseased ones. The specificity metric is defined in Equation 8 as follows:


$$\begin{array}{l} {\text{Specificity}}_{i}=\frac{TN_{i}}{FP_{i}+TN_{i}} \end{array}$$ (8)

#### 4.6.4 Precision

Precision represents the proportion of correct predictions of positive instances among the total cases that the model predicts as positive. If the precision of a model is very high, then that means the model hardly makes mistakes. This is mostly critical in situations where a negative case being wrongly classified as positive results in a significant loss. The precision metric is defined in Equation 9 as follows:


$$\begin{array}{l} {\text{Precision}}_{i}=\frac{TP_{i}}{TP_{i}+FP_{i}} \end{array}$$ (9)

#### 4.6.5 F1-score

F1-Score is calculated as the average of precision and recall, thus being an aggregate score of how accurate the model is. Hence, by its very nature, it is a trade-off between these two measures, penalizing models with too many false positives and models with too many false negatives. That is why F1 is a particularly crucial score for problems with class imbalance. A high F1-score will show that the model is well-balanced in both precision and recall. The F1-score metric is defined in Equation 10 as follows:


$$\begin{array}{l} {\text{F1-Score}}_{i}=\frac{2TP_{i}}{2TP_{i}+FP_{i}+FN_{i}} \end{array}$$ (10)

The metrics were first calculated per class and then averaged using macro averaging, which gives equal weight to each class, irrespective of the class frequencies in the data set. Overall, these criteria provide a reliable basis to evaluate the classification accuracy and clinical applicability of the proposed model.

## 5 Experimental results

### 5.1 Model performance: training and validation

**Figure 6:**
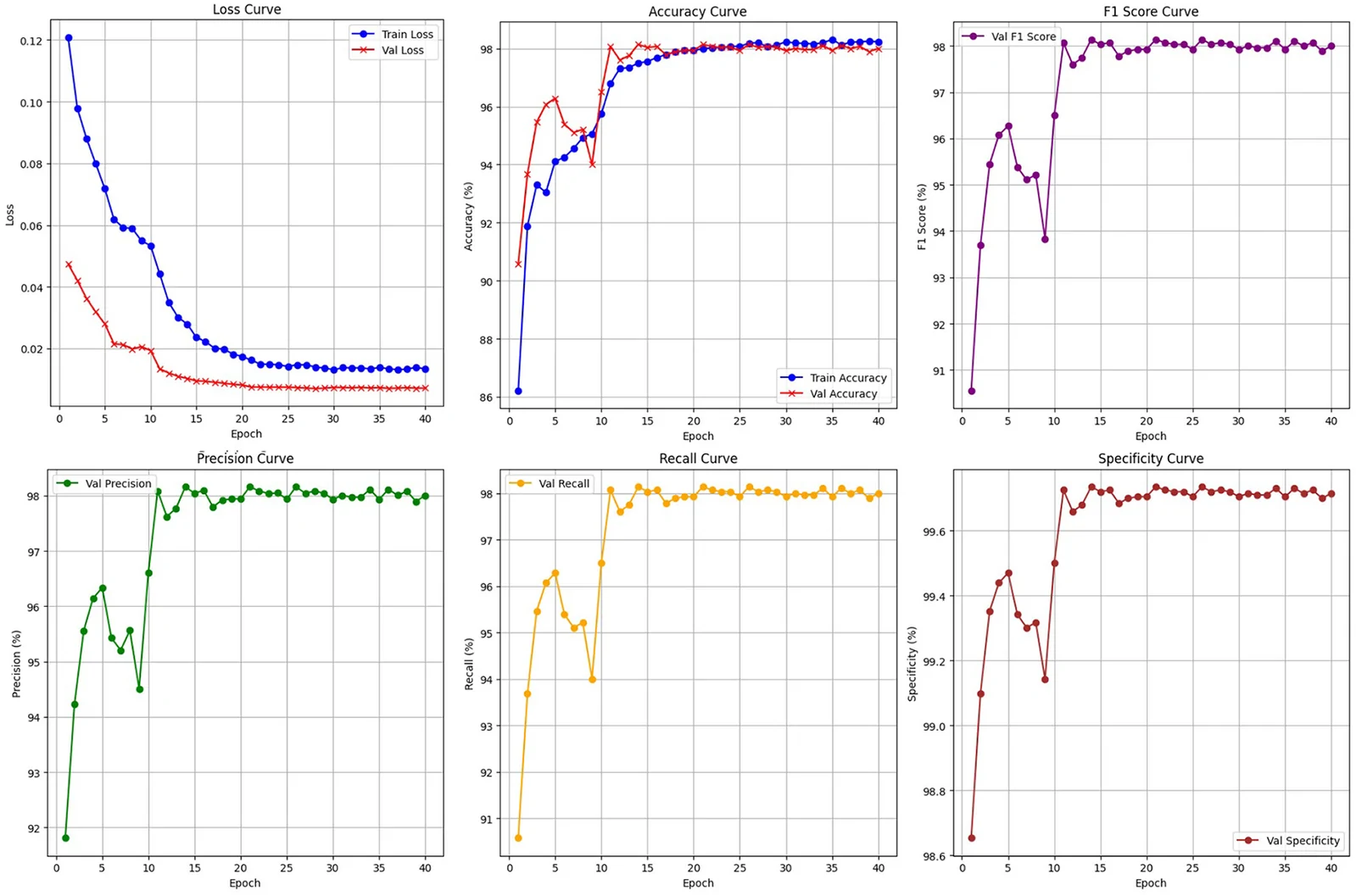
Learning curves of the proposed YOLOv11-based OCT classification model, showing training and validation loss, accuracy, F1-score, precision, recall, and specificity over 40 epochs.

The performance of the proposed model shows steady improvement in the 40-epoch training process. Figure 6 shows the training and validation loss curves, both of which were on a constant downward trend. The training loss decreased significantly from 0.12 to 0.014, and the validation loss also decreased from 0.05 to 0.008, hence proving that effective learning was taking place and there was no overfitting.

Furthermore, the model's accuracy showed significant gains. The training accuracy increased from 86.21% to 98.23%, while the validation accuracy increased from 90.57% to 98.00%. This clearly shows that there is a high level of generalization. Precision and recall metrics are also constantly increasing while getting stabilized at the last epoch. Validation scores reached 0.9800 for F1-score, precision, recall, and 0.9971 for specificity.

The selected epoch wise performance is summarized in Table 5, indicating significant improvements during the course of training. The best validation accuracy at 98.14% was achieved at epoch 34; consistently high values persisted over subsequent epochs, confirming model robustness.

**Table 5:** Evolution of training and validation metrics across epochs.

Epoch	Train loss	Train Acc	Val loss	Val Acc	F1-score	Precision	Recall	Specificity
1	0.120	86.21%	0.055	90.57%	0.9055	0.9181	0.9057	0.9865
10	0.040	95.74%	0.020	96.50%	0.9650	0.9660	0.9650	0.9950
20	0.020	97.94%	0.012	97.93%	0.9793	0.9794	0.9793	0.9970
31	0.015	98.19%	0.009	98.00%	0.9800	0.9800	0.9800	0.9971
40	0.014	98.23%	0.008	98.00%	0.9800	0.9800	0.9800	0.9971

### 5.2 Evaluation on test sets

The performance of the YOLOv11-based classification model was tested with a test dataset composed of 2,800 retinal OCT scans equally representing the 8 disease classes (350 samples per class). Performances were evaluated using several well established evaluation metrics which include accuracy, precision, recall, F1-score, and specificity. In this context, the model performed well, achieving 98.00% micro averaged accuracy while the macro averaged precision, recall, and F1-score were recorded as 0.98, with the macro averaged specificity of 0.9971. The above findings indicate the robustness and generalization feature of the proposed method.

Analysis of the individual classes reveals improved performance of the model. The model performed perfectly well for the MH, CSR, AMD, and DR classes achieving an F1-score of 1.00 for each of the classes. This illustrates that the model is very effective in feature extraction. In addition, the model is able to make accurate decisions for these diseases. For the difficult classes of the OCT images such as CNV and DRUSEN, the model performed extremely well with an F1-score of 0.95. The reduction in the F1-score for these classes is due to the visual similarities within the OCT images.

**Figure 7:**
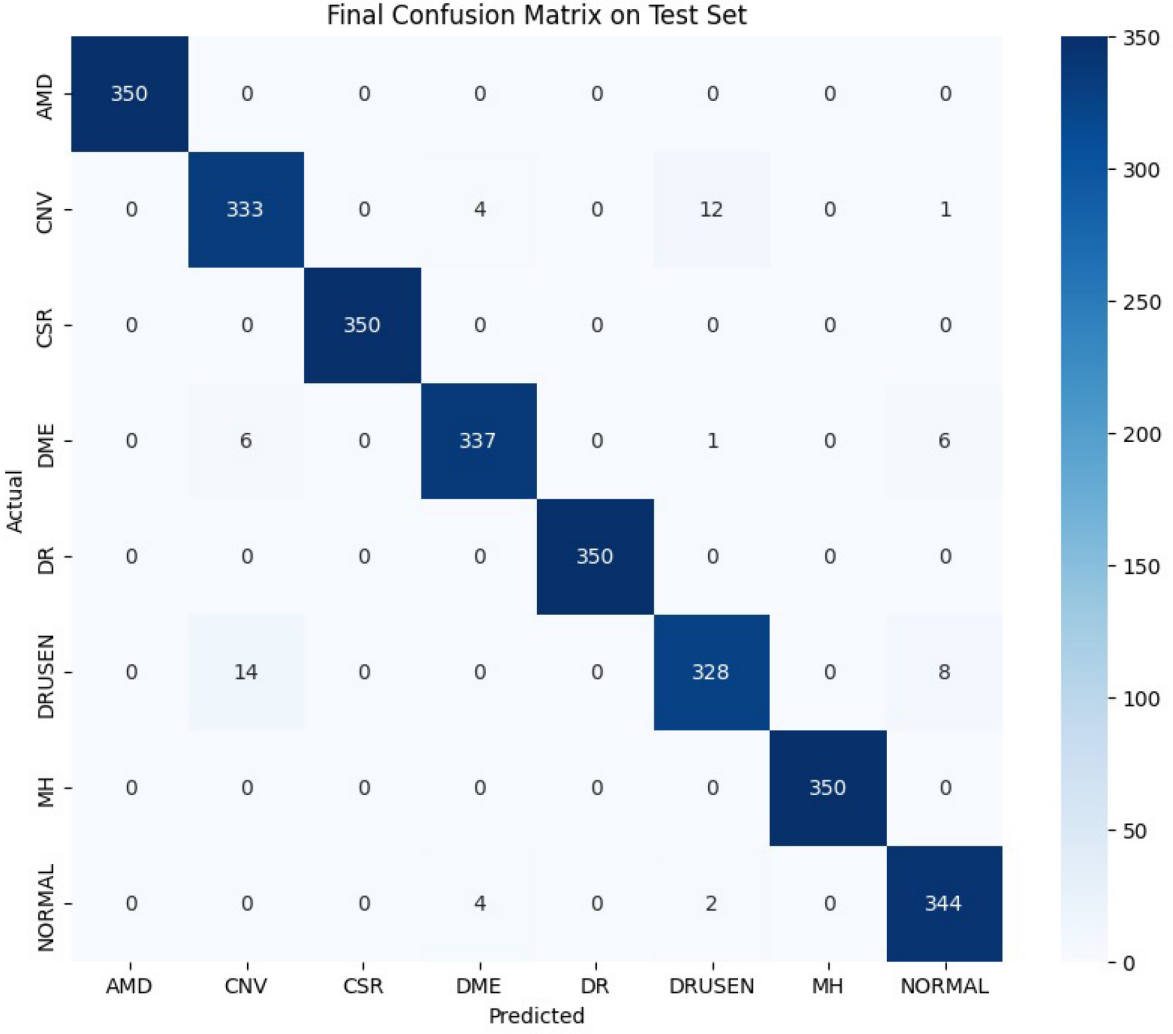
Confusion matrix of the proposed YOLOv11-based OCT classification model on the test set. Most samples are correctly classified along the main diagonal, with only a small number of misclassifications observed between a few retinal disease categories.

The confusion matrix illustrated in Figure 7 shows strong diagonal dominance with very few misclassified instances. Misclassifications are mainly between

- CNV and DRUSEN.
- DME and NORMAL classes.

The model still achieved high overall recall (macro average 0.98) and precision (macro average 0.98) regardless of these problems, thus providing clinical reliability.

#### 5.2.1 Error analysis and representative failure cases

**Figure 8:**
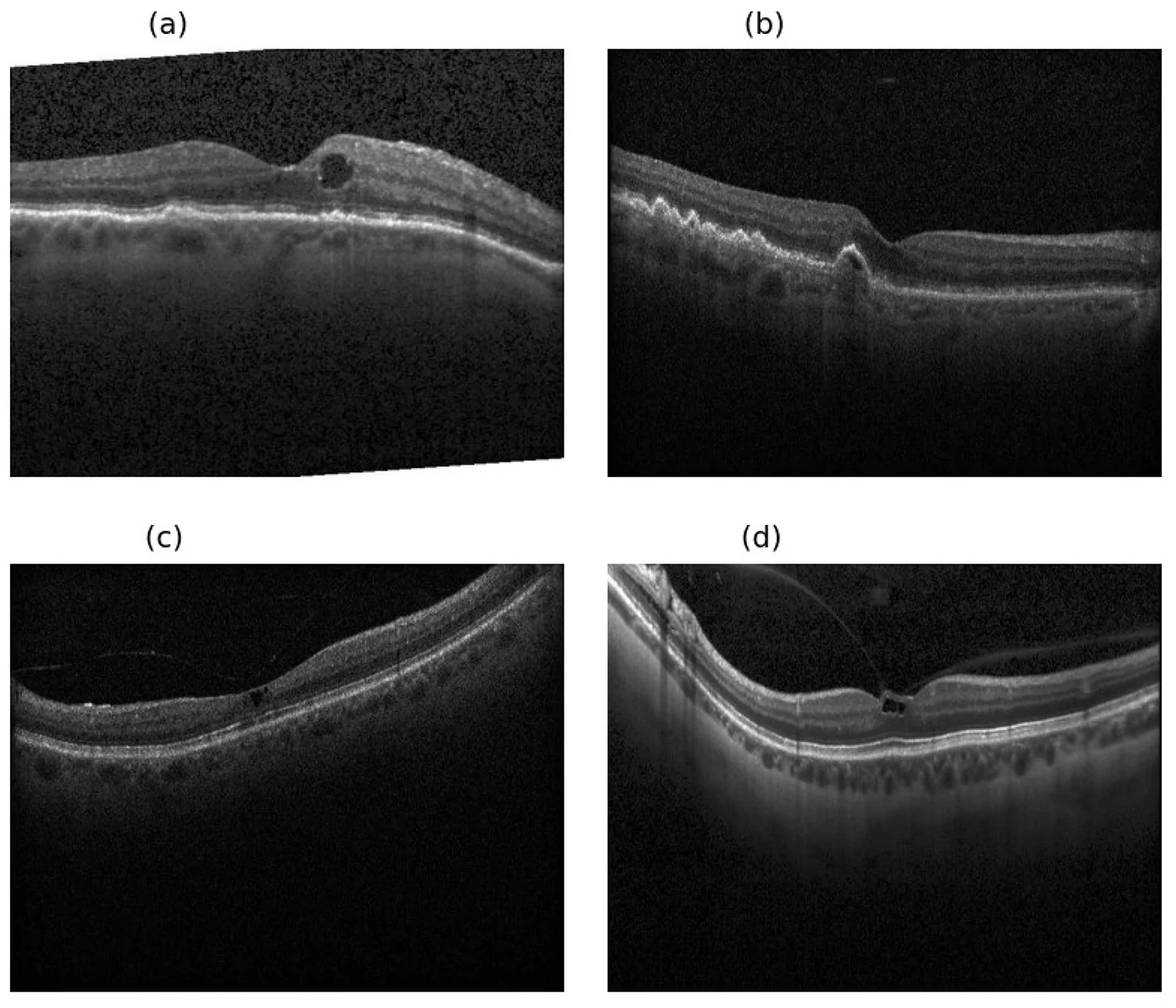
Representative failure cases of the proposed YOLOv11-based OCT classification model. The examples show challenging misclassification patterns identified from the test-set confusion matrix, including CNV–DRUSEN and DME–NORMAL confusions. The first and second labels indicate the ground-truth class and predicted class, respectively. These cases demonstrate the difficulty in distinguishing retinal conditions with overlapping structural characteristics in OCT images. **(a)** CNV → DRUSEN. **(b)** DRUSEN → CNV. **(c)** DME → NORMAL. **(d)** NORMAL → DME.

To further investigate the misclassification behavior of the proposed model, representative failure cases were analyzed from the test-set predictions. The major confusion patterns identified from the confusion matrix were CNV-DRUSEN and DME-NORMAL. Figure 8 shows representative examples of such difficult cases. The model misclassified 14 CNV samples as DRUSEN and 11 DRUSEN samples as CNV, which is a reflection of the difficulty to distinguish the diseases with similar structural characteristics of the retina. Similarly, 6 DME samples were predicted as NORMAL and 5 NORMAL samples predicted as DME. These errors may be attributed to subtle pathological variations and overlapping morphological appearances in OCT scans. The failure analysis demonstrates that while the proposed framework achieves strong overall performance, some visually similar retinal conditions remain challenging for automated classification.

Overall, the proposed model performs well in the classification of retinal OCT images. The detailed classification report of the test set is provided in Table 6.

**Table 6:** Classification report on the test set showing per-class performance metrics of the proposed YOLOv11-based model.

Class	Precision	Recall	F1-score	Specificity
AMD	1.00	1.00	1.00	1.00
CNV	0.94	0.95	0.95	0.99
CSR	1.00	1.00	1.00	1.00
DME	0.98	0.96	0.97	0.99
DR	1.00	1.00	1.00	1.00
Drusen	0.96	0.94	0.95	0.99
MH	1.00	1.00	1.00	1.00
Normal	0.96	0.98	0.97	0.99
Macro Avg.	0.98	0.98	0.98	0.997
Weighted Avg.	0.98	0.98	0.98	0.997

### 5.3 Statistical analysis

To confirm that the improvements achieved by the proposed method were not the result of random variation, a comprehensive statistical analysis was carried out. A two-way ANOVA (Fisher, 1970) without replication was initially employed to assess the overall significance of differences among models and disease categories. In order to detect pairwise differences, a Tukey HSD test was further conducted in order to compare the proposed technique to the baseline techniques in relation to all the criteria.

#### 5.3.1 ANOVA results

The predictive capacity of the model has been thoroughly analyzed using two-way ANOVA without replication whereby the performance of the model has been compared to that of two other benchmark models (Laouarem et al., 2024; Pan et al., 2025). The performance metrics involved in the analysis were the five common metrics which include accuracy, recall, precision, F1-Score and specificity with class-wise measurements being considered in the eight disease categories. This structure allowed us to assess both differences between the models and the effect of disease type on performance.

ANOVA findings as shown in Table 7 demonstrate that the method type used has a significant impact on some performance metrics. A statistically significant difference in accuracy was found (F = 5.58, *p* = 0.016), which indicates that the classification method has a significant effect on accuracy. Also, there was a significant difference in precision (F = 5.75, *p* = 0.049), indicating a significant effect on the prediction of positive cases. The largest difference among the method effects was observed in F1-score (F = 11.80, *p* = 0.001), which signifies a significant difference in the balance between recall and precision. There was no significant difference in recall (F = 2.30, p = 0.137), indicating that the methods have similar sensitivity. Also, there was no significant difference in specificity (F = 0.94, *p* = 0.412).

**Table 7:** Results of the two-way ANOVA assessing the effects of disease class and classification method on model performance metrics.

Metric	Source of variation	F-value	*P*-value	Significance
Accuracy	Disease class	3.48	0.022	Yes
Accuracy	Method	5.58	0.016	Yes
Precision	Disease class	20.17	<0.001	Yes
Precision	Method	5.75	0.049	Yes
Recall	Disease class	20.83	<0.001	Yes
Recall	Method	2.30	0.137	No
F1-Score	Disease class	77.72	<0.001	Yes
F1-Score	Method	11.80	0.001	Yes
Specificity	Disease class	6.90	0.001	Yes
Specificity	Method	0.94	0.412	No

The ANOVA test further verified that classification performance differs significantly between different disease classes. This is true for accuracy (*F* = 3.48, *p* = 0.022), precision (*F* = 20.17, *p* < 0.001), recall (*F* = 20.83, *p* < 0.001), and F1-score (*F* = 77.72, *p* < 0.001). Specificity also showed a statistically significant difference across disease classes (*F* = 6.90, *p* = 0.001).

#### 5.3.2 Tukey's HSD post-hoc analysis

To examine these pairwise differences in more detail, a Tukey HSD *post hoc* comparison was done and its findings are displayed in Table 8. All the Tukey comparisons use the macro averaged per class metrics as the basis. This analysis confirmed that proposed model achieves statistically comparable or improved performance relative to baseline architectures (Laouarem et al., 2024), particularly in precision and F1-score, while maintaining consistent accuracy, recall, and specificity. For the remaining metrics, including accuracy, recall and specificity, the results indicate that the proposed model performs at a level statistically equivalent to the strongest existing benchmarks, such as Laouarem et al. (2024) and Pan et al. (2025), as the mean differences did not exceed the calculated HSD critical thresholds. These findings demonstrate that the proposed YOLOv11-based framework is both robust and highly competitive.

**Table 8:** Tukey's HSD post-hoc comparison of the proposed YOLOv11-based model against benchmark methods at 95% confidence level.

Metric	Proposed Avg (%)	Compared model	Compared (%)	Mean diff.	HSD	Significance
Accuracy	98.43	HTC-Retina (Hybrid) (Laouarem et al., 2024)	97.00	+1.43	2.06	No significant diff.
Accuracy	98.43	Lightweight CNN + Transformer (Pan et al., 2025)	99.63	-1.20	2.06	No significant diff.
Precision	98.25	HTC-Retina (Hybrid) (Laouarem et al., 2024)	96.70	+1.56	1.30	**Proposed better**
Precision	98.25	Lightweight CNN + Transformer (Pan et al., 2025)	98.02	+0.23	1.30	No significant diff.
Recall	97.88	HTC-Retina (Hybrid) (Laouarem et al., 2024)	97.00	+0.88	1.33	No significant diff.
Recall	97.88	Lightweight CNN + Transformer (Pan et al., 2025)	98.00	-0.12	1.33	No significant diff.
Specificity	99.70	HTC-Retina (Hybrid) (Laouarem et al., 2024)	99.57	+0.13	0.31	No significant diff.
Specificity	99.70	Lightweight CNN + Transformer (Pan et al., 2025)	99.71	-0.01	0.31	No significant diff.
F1-Score	98.00	HTC-Retina (Hybrid) (Laouarem et al., 2024)	97.00	+1.00	0.62	**Proposed better**
F1-Score	98.00	Lightweight CNN + Transformer (Pan et al., 2025)	98.00	+0.00	0.62	No significant diff.

The HSD was calculated at a 95% confidence level using the formula presented in Equation 11:


$$\begin{array}{l} HSD=q_{\alpha }\times \sqrt{\frac{MSE}{n}} \end{array}$$ (11)

Here, *q*_α_ is the critical value from the standardized range distribution. *MSE* is the mean squared error (i. e. variance within group), which is based on ANOVA. *n* is the number of observations per group. Pairwise differences between group means were considered to be statistically significant if the absolute mean differences were greater than the calculated HSD values.

In summary, the results of both ANOVA and Tukey tests provide a solid statistical basis to validate the methodology. Moreover, the analysis shows a significant improvement over one of the baselines while being statistically equivalent to the other baseline.

### 5.4 Model complexity analysis

To fully evaluate the computational efficiency and deployability of the proposed model, the analysis considers several computational metrics such as Floating Point Operations (FLOPs), number of parameters, model size, inference latency, throughput (FPS), and peak GPU memory usage. The proposed model is compared with representative CNN and transformer based classification architectures under identical input conditions for fair evaluation of computational characteristics.

Table 9 presents the computational complexity metrics of the proposed model and the baseline architectures. THOP (PyTorch OpCounter) library was used to compute FLOPs and parameter counts, to keep all evaluated models consistent. The proposed model was trained on higher resolution OCT images of size 450 × 450 to capture detailed retinal structures; however, computational comparisons were made on a standard input resolution of 224 × 224 × 3 to ensure a fair comparison against benchmark architectures. In addition, inference latency, FPS, and max GPU memory consumption are tested under the same GPU environment to verify the deployment efficiency in practice.

**Table 9:** Comparison of model complexity and inference efficiency between the proposed YOLOv11+PSF+SE+Head model and CNN and Transformer architectures.

Model	Parameters (M)	GFLOPs	Size (MB)	Inference (ms)	FPS	Peak GPU memory (GB)
VGG16	138.36	15.47	527.80	8.80	110.97	1.293
ViT-Base-16	86.38	16.85	346.00	14.83	47.21	0.411
ResNet-101	44.55	7.87	170.56	11.33	87.93	0.597
ConvNeXt-Tiny	28.57	4.46	114.00	12.89	85.76	0.806
Swin-Poly Transformer (He et al., 2023)	27.50	4.50	–	12.60	–	–
ResNet-50	25.56	4.13	97.81	8.19	176.06	0.661
ResNet-34	21.80	3.68	83.30	3.88	243.85	0.679
EfficientNet-B4	19.22	1.50	74.78	17.45	64.44	1.467
DenseNet-121	7.98	2.90	31.06	17.82	67.97	0.692
MobileNetV2	3.51	0.33	13.63	5.18	200.44	0.091
YOLOv11n-Classifier	2.81	0.20	10.89	5.31	192.41	0.817
YOLOv8n-Classifier	2.72	0.21	10.48	4.69	303.68	0.804
**Proposed YOLOv11+PSF+SE+Head**	**9.06**	**14.25**	**34.74**	**5.84**	**115.99**	**0.854**

As in Table 9, the proposed YOLOv11+PSF+SE+Head framework has 14.25 GFLOPs and 9.06 million parameters. The proposed architecture introduces additional computational requirements compared with lightweight models such as MobileNetV2 and YOLOv11n-Classifier because of the incorporation of the Progressive Spatial Fusion (PSF) module, attention refinement modules and the modified classification head. These extra operations are intentionally incorporated to facilitate multi-scale feature aggregation and subtle retinal abnormalities discrimination while preserving practical feasibility of deployment. The number of parameters is still much lower than high capacity architectures such as ResNet-101 (44.549 M) and VGG16 (138.358 M) which indicates a good trade-off between better feature representation and computational cost.

From the perspective of practical deployment, the proposed model maintains a compact size of 34.74 MB. Under a CUDA-supported GPU environment, the proposed framework achieves an average inference latency of 5.84 ms per image. Additional evaluation using throughput and memory consumption demonstrates that the model achieves 115.99 FPS with a peak GPU memory requirement of 0.854 GB. Although the proposed architecture introduces additional computational operations compared with lightweight networks, these results indicate that it maintains practical inference efficiency while providing improved feature representation capability for OCT classification.

Overall, the computational analysis shows that the proposed YOLOv11+PSF+SE+Head method has a good performance-complexity trade-off. Although the integration of PSF and attention mechanisms increases computational requirements, the model maintains practical inference speed, moderate memory consumption, and efficient deployment characteristics. These properties support its potential application in real-time OCT-based retinal disease screening scenarios.

### 5.5 Grad-CAM based interpretability and retinal layer correlation study

The interpretability of the proposed model was explored using Grad-CAM. Grad-CAM offers visual explanations by highlighting the regions of the images that are used to make classification decisions. To analyze whether the model attends to anatomically relevant regions, the obtained activation maps were qualitatively compared to disease-associated retinal structures and pathological patterns reported in ophthalmic literature.

**Figure 9:**
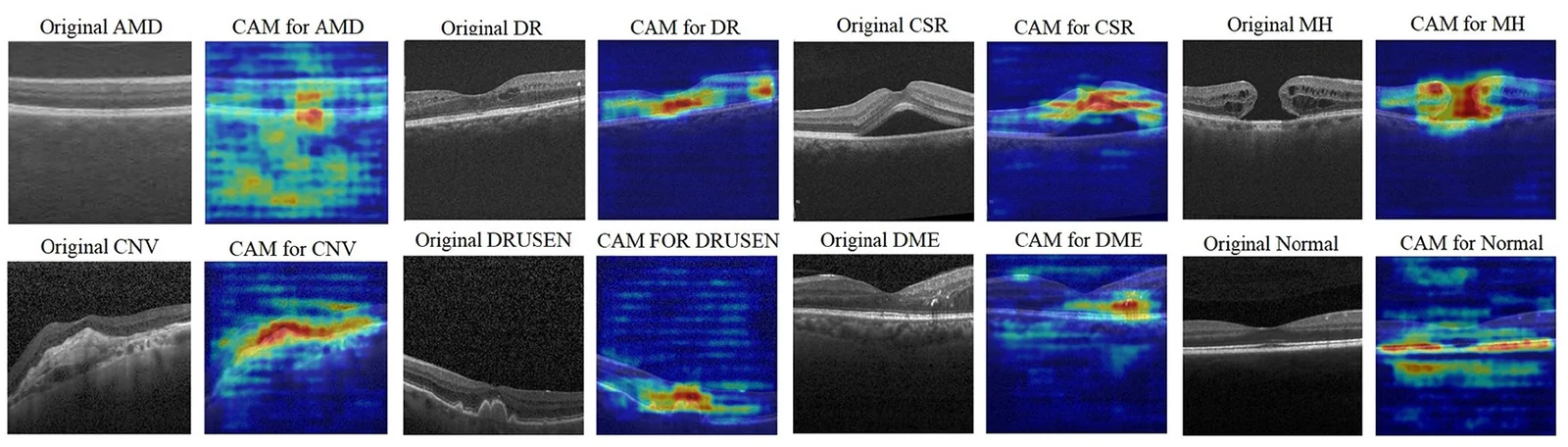
Grad-CAM results for all eight retinal OCT classes. Each pair shows the original OCT scan **(left)** and the corresponding Grad-CAM heatmap **(right)**, highlighting image regions contributing to the model prediction.

Figure 9 shows the Grad-CAM outputs for all eight retinal classes. Each row gives one original OCT image (left) with its corresponding Grad-CAM heatmap (right). Red and yellow areas imply high model attention, indicating where the network focuses its attention while identifying disease-related patterns.

Comparison between disease-associated retinal features and observed Grad-CAM activation regions is presented in Table 10. The retinal structures and pathological characteristics considered for each disease category were based on previously reported OCT biomarkers and clinical imaging findings. The analysis indicates a qualitative correspondence between the regions emphasized by the model and clinically relevant retinal structures associated with each disease category.

**Table 10:** Qualitative comparison between literature-reported disease-associated OCT biomarkers and corresponding Grad-CAM activation regions of the proposed model. The analysis provides insight into whether model attention corresponds with known retinal structural patterns; it does not represent quantitative lesion localization validation. ONL, Outer Nuclear Layer; RPE, Retinal Pigment Epithelium; RNFL, Retinal Nerve Fiber Layer.

Class	Disease-associated OCT features (literature-based)	Qualitative correspondence between Grad-CAM activation and reported OCT features	Match
AMD	RNFL, ONL, disruption near RPE (Sadda et al., 2018; Ferris III et al., 2013)	ONL-RPE region	Qualitative correspondence observed
CNV	RNFL, ONL, RPE (sub-RPE fluid) (Kermany et al., 2018; Schmidt-Erfurth et al., 2014)	RPE and subretinal area	Qualitative correspondence observed
CSR	RPE, subretinal fluid space (Wong et al., 2016)	Subretinal fluid space	Qualitative correspondence observed
DME	RPE, RNFL, cystoid spaces (Otani et al., 1999)	Inner retina (cystoid changes)	Qualitative correspondence observed
DR	RNFL, ONL, intraretinal thickening (Wilkinson et al., 2003)	Middle retinal layers	Qualitative correspondence observed
Drusen	Between RPE and Bruch's membrane (Schlanitz et al., 2015)	RPE–Bruch's membrane interface	Qualitative correspondence observed
MH	Foveal depression and thinning (Duker et al., 2013)	Foveal gap (thinning)	Qualitative correspondence observed
Normal	Uniform layer boundaries (RNFL, RPE) (Drexler and Fujimoto, 2008; Spaide and Curcio, 2011)	Low and diffuse activation	Qualitative correspondence observed

Layer-specific attention patterns observed in Grad-CAM heatmaps showed qualitative correspondence with disease-associated retinal locations, such as the subretinal space in CSR and the foveal region in MH. When it comes to healthy images, the heatmaps show a rather diffuse or even almost non-existent activation, which implies that the model is very confident and quite certain that the identified scans are normal. However, very distinct and tightly localized zones of activation are seen in pathological images, indicating that the model captures relevant structural and pathological cues associated with disease classification. These Grad-CAM visualizations provide insights into the learned decision-making patterns of the model and improve the transparency of its predictions.

However, since the OCT-C8 dataset provides image-level labels without pixel-level lesion annotations, the Grad-CAM analysis is interpreted as a qualitative explanation method rather than a quantitative localization evaluation. The obtained activation patterns provide insights into model decision-making, while more detailed annotation-based evaluation remains beyond the scope of the current study. Furthermore, expert ophthalmologist assessment of Grad-CAM activation patterns was not performed in this study. Such evaluation would provide valuable additional validation of model explanations. Moreover, the absence of lesion-level annotations and expert-defined localization boundaries in OCT-C8 prevents quantitative assessment of activation overlap with pathological regions. Future studies will include ophthalmologist-assisted evaluation using expert-annotated OCT datasets.

### 5.6 Comparison with existing OCT classification methods

The performance of the proposed YOLOv11+PSF+SE+Head framework was compared with previously reported OCT-based retinal disease classification methods to position its performance within the current research landscape. The compared approaches were selected based on their evaluation on the OCT-C8 benchmark dataset and the same eight retinal disease categories, providing a meaningful reference for assessing the effectiveness of the proposed framework. However, since these methods were developed independently, variations in implementation details, including preprocessing procedures, augmentation strategies, optimization parameters, and training configurations, may exist across studies. Therefore, the comparisons presented in Table 11 should be interpreted as benchmark-level comparisons rather than strictly controlled head-to-head evaluations. To ensure fair assessment within this work, all baseline comparisons, ablation studies, and proposed model evaluations were conducted using the same OCT-C8 dataset partitions, preprocessing pipeline, training strategy, and evaluation metrics. The comparative performance of the proposed framework with existing OCT classification approaches is summarized in Table 11.

**Table 11:** Comparison of retinal disease classification models on OCT-C8 dataset. The proposed model is benchmarked against state-of-the-art transformer and hybrid architectures.

Model	Accuracy	F1-score	Recall	Precision	Specificity
Swin-Poly Transformer (He et al., 2023)	97.12%	97.10%	97.13%	97.13%	–
HTC-Retina (Hybrid) (Laouarem et al., 2024)	97.00%	97.01%	97.00%	97.04%	99.56%
ResNet34 (Modified) (Karthik and Mahadevappa, 2023)	92.40%	92.00%	92.00%	93.00%	–
ResNet50 (Modified) (Karthik and Mahadevappa, 2023)	90.30%	91.00%	91.00%	91.00%	–
ResNet101 (Modified) (Karthik and Mahadevappa, 2023)	86.10%	86.00%	86.00%	86.00%	–
Lightweight CNN-Transformer (Pan et al., 2025)	98.50%	97.99%	97.99%	98.02%	99.71%
**Proposed model**	**98.00%**	**98.00%**	**98.00%**	**98.00%**	**99.70%**

As shown in Table 11, the proposed framework achieves competitive classification performance compared with previously reported OCT classification approaches. The improved performance is achieved through the integration of the PSF module, SE attention mechanism, and customized classification head, which enhance multi-scale feature representation and discriminative feature learning. Although the proposed framework introduces additional computational cost compared with compact architectures, the achieved balance between classification performance and computational complexity demonstrates its suitability for OCT-based retinal disease classification.

### 5.7 Ablation study of proposed model

To understand the contribution of the major components in the proposed architecture, an extensive ablation study was performed using different model configurations based on three independent runs. The ablation study was designed to evaluate the progressive contribution of the major architectural components introduced into the YOLOv11 backbone. Starting from the baseline YOLOv11 architecture, the effects of the modified classification head, Progressive Spatial Fusion (PSF) module, and SE-based dual-attention refinement mechanism were analyzed through incremental model configurations. Since the proposed SE-based dual-attention refinement mechanism integrates SE channel attention and spatial attention refinement as a unified block, the attention contribution is evaluated through the combined refinement pathway rather than by isolating spatial attention as an independent module. Starting from the YOLOv11 Base architecture, three additional components were integrated step-by-step: the SE-based dual-attention refinement block, the PSF module, and finally, the newly designed custom classification head. The performance of each variant was evaluated for accuracy, F1-score, recall, precision, and specificity. Also the mean standard deviation for all the metrics were taken to indicate the consistency and robustness of each component. The results of this study are presented in Table 12.

**Table 12:** Ablation study of the proposed YOLOv11+PSF+SE+Head model, showing the progressive contribution of major architectural components, including Progressive Spatial Fusion (PSF), attention refinement, and the modified classification head. Results are reported as mean ± standard deviation over three independent runs.

Variant	Accuracy	F1	Precision	Recall	Specificity
YOLOv11 Base	0.952 ± 0.0014	0.952 ± 0.0013	0.953 ± 0.0015	0.952 ± 0.0014	0.993 ± 0.0002
YOLOv11 + Head	0.954 ± 0.0049	0.954 ± 0.0050	0.955 ± 0.0047	0.954 ± 0.0049	0.993 ± 0.0007
YOLOv11 + PSF	0.957 ± 0.0023	0.957 ± 0.0022	0.957 ± 0.0026	0.957 ± 0.0023	0.994 ± 0.0003
YOLOv11 + PSF + Head	0.972 ± 0.0029	0.972 ± 0.0030	0.973 ± 0.0026	0.972 ± 0.0029	0.995 ± 0.0004
YOLOv11 + SE	0.951 ± 0.0064	0.951 ± 0.0065	0.952 ± 0.0062	0.951 ± 0.0064	0.993 ± 0.0009
YOLOv11 + SE + Head	0.958 ± 0.0039	0.958 ± 0.0040	0.958 ± 0.0036	0.958 ± 0.0039	0.993 ± 0.0006
YOLOv11 + SE + PSF	0.964 ± 0.0008	0.964 ± 0.0009	0.965 ± 0.0009	0.964 ± 0.0008	0.993 ± 0.0001
**Final proposed model**	**0.980** **±0.0034**	**0.980** **±0.0035**	**0.980** **±0.0032**	**0.980** **±0.0034**	**0.997** **±0.0005**

The YOLOv11 Base model serves as our baseline, achieving a respectable accuracy of 95.2% ± 0.13%. The first modification involves replacing the conventional detection head with our proposed custom classification head. This modification, represented by YOLOv11+Head, resulted in a slight increase in performance, with accuracy improving to 95.4% ± 0.49%. This shows that the modified head provides a marginal but positive impact on feature discrimination.

We then introduced the PSF module, which extracts multi-scale contextual information. The YOLOv11+PSF model achieved an accuracy of 95.7% ± 0.23%, which clearly outperforms the baseline model. With the inclusion of both the PSF module and our custom classification head into the base model, a remarkable performance leap occurred with YOLOv11+PSF+Head at an accuracy of 97.2% ± 0.29%. A strong combined effect of the PSF's multi-scale feature fusion and the attention head's improved feature recognition was consistently observed across multiple runs.

The effect of the SE-based dual-attention refinement block was also evaluated. The YOLOv11+SE attention refinement configuration reached an accuracy of 95.1% ± 0.64%, which is slightly lower than the baseline model. However, when combined with the modified classification head (YOLOv11+SE+Head), its accuracy increased to 95.8% ± 0.39%, demonstrating the complementary contribution of attention refinement and improved classification capability. We observe from this that channel wise feature recalibration offered by the SE block is utilized more effectively when combined with our proposed attention mechanism.

The two-stage optimization strategy was not included as an architectural ablation component because it represents an optimization procedure rather than a structural modification of the network. The first stage performs classifier adaptation, while the second stage performs end-to-end fine-tuning of the complete framework. Therefore, its role is discussed separately in the training strategy section.

The most significant improvements were observed when all components were put together. The YOLOv11+SE+PSF model, incorporating both the SE block and the PSF module, achieved an accuracy of 96.4% ± 0.08%. The final proposed model, the YOLOv11+PSF+SE+Head model that incorporates all the three components, had the best performance and recorded an overall accuracy of 98.0 ± 0.34%. This final model also exhibited an excellent specificity (0.997 ± 0.00049) which highlights its superior ability to correctly identify negative cases.

This ablation study confirms that each architectural component contributes progressively to performance improvement, with the combined integration of PSF, dual-attention refinement, and the customized classification head yielding the highest overall diagnostic accuracy.

## 6 Cross dataset generalization

To test the ability of the proposed YOLOv11+PSF+SE+Head setup to generalize beyond the OCT-C8 dataset, cross-dataset validation was carried out on the publicly available Kaggle version of the Kermany OCT2017 Version 2 dataset (Mooney, 2018), which was collected independently from the OCT-C8 dataset. This dataset was never used for training, validation, or model selection and consists of OCT images collected under different dataset conditions and imaging distributions compared with OCT-C8. Testing on this independent dataset enables assessment of the resilience of the proposed model under domain shift and also verify that the learned representations are not overfitted to the OCT-C8 dataset. The Kermany OCT2017 Version 2 dataset contains four retinal disease categories (CNV, DME, DRUSEN, and NORMAL), which represent a subset of the eight categories available in the OCT-C8 dataset. Therefore, external evaluation was performed using only the overlapping classes between the two datasets. No additional training, fine-tuning, class adaptation, or parameter optimization was performed using the external dataset. Although this evaluation does not cover all disease categories targeted by the proposed eight-class framework, it provides an independent assessment of feature generalization and robustness under dataset variation without additional training or fine-tuning.

**Figure 10:**
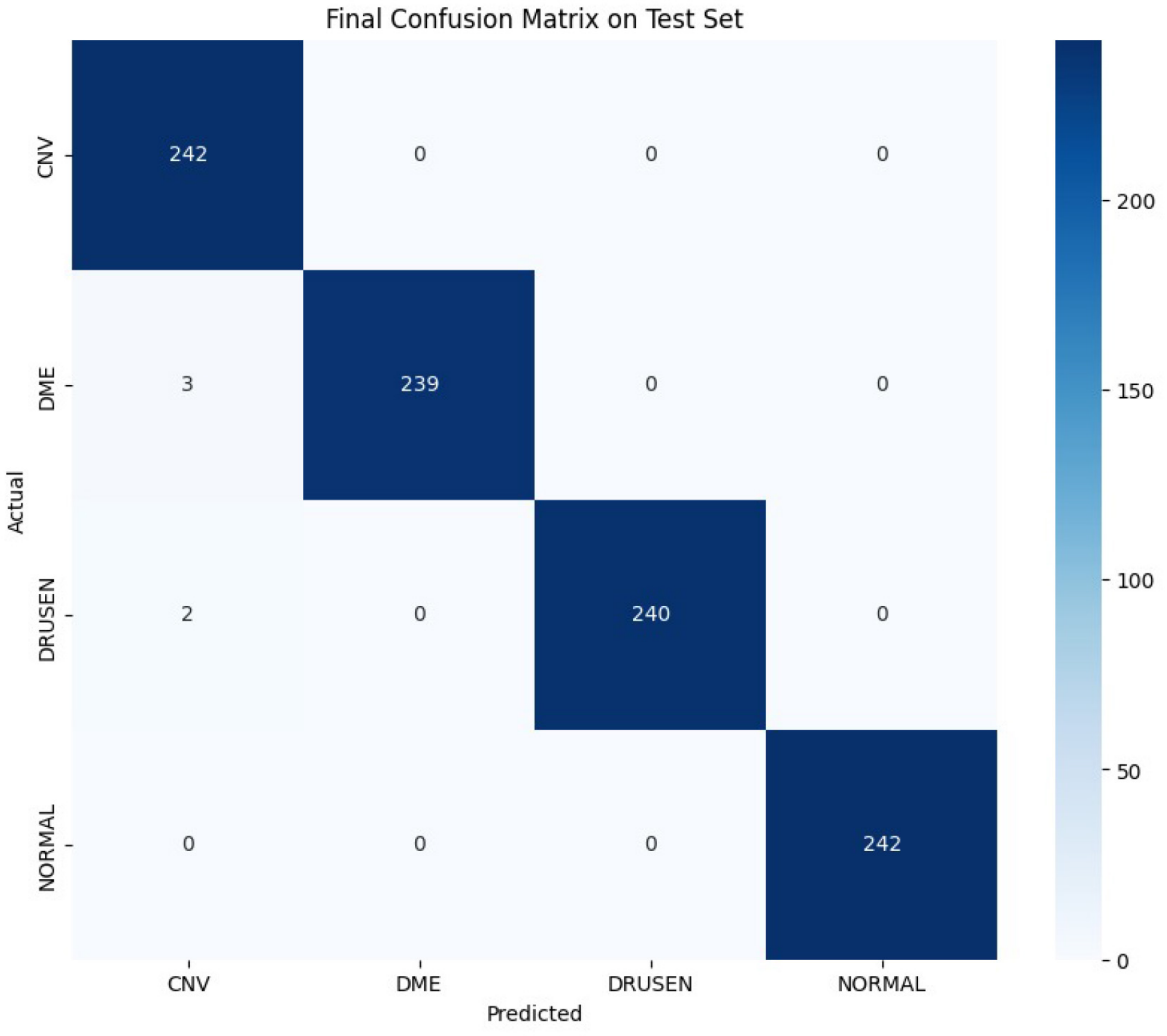
Confusion matrix of the proposed YOLOv11+PSF+SE+Head model on the Kermany OCT2017 Version 2 test split, illustrating class-wise prediction performance across CNV, DME, Drusen, and Normal categories.

The YOLOv11+PSF+SE+Head system achieved an overall classification accuracy of 99.5% on the Kermany OCT2017 Version 2 test split. The class-wise performance has been consistently very good, with recall, precision, and F1-score values ranging from 0.99 to 1.00 for CNV, DME, Drusen, and Normal classes, all of which have been summarized in Table 13. In addition, only five misclassifications out of 968 test samples have been detected per the confusion matrix in Figure 10, and Normal images have not been misclassified at all. These results indicate that the proposed model demonstrates promising cross-dataset generalization capability under the domain variation between OCT-C8 and the external Kermany OCT2017 dataset

**Table 13:** Classification report on the publicly available Kaggle version of the Kermany OCT2017 Version 2 test split showing per-class performance metrics of the proposed YOLOv11+PSF+SE+Head model.

Class	Precision	Recall	F1	Specificity
CNV	0.98	1.00	0.99	0.99
DME	1.00	0.99	0.99	1.00
Drusen	1.00	0.99	1.00	1.00
Normal	1.00	1.00	1.00	1.00
Macro Avg.	0.99	0.99	0.99	0.998
Weighted Avg.	0.99	0.99	0.99	0.998

## 7 Discussion

Over the past decade, the field of OCT based retinal disease classification has drastically changed. The change was from binary decision systems that was trained on very small datasets (Sadda et al., 2006; Liu et al., 2011; Helmy and Atta Allah, 2013) to very complex multiclass systems. At first, the four class setting studies performed very well with high accuracy (over 96%) being reported in some papers (Kermany et al., 2018; Raghavendra et al., 2018; Tsuji et al., 2020; Das et al., 2021). Still, a number of these methods seemed to focus mainly on accuracy with only a few addressing the class-wise balance and robustness across diverse evaluation metrics. More recent studies have extended the scope of OCT classification to multi-class problems. For example, Alqudah (2020) proposed a method called AOCT-NET for five class classification, which achieved 95.30% accuracy. Similarly Laouarem et al. (2024) presented HTCRetina which is a hybrid CNN-Transformer model achieving 97.00% accuracy on an eight-class OCT dataset. Despite these advances, achieving simultaneous improvements in accuracy, class-wise balance, and computational efficiency remains challenging as problem complexity increases.

Modern compact networks also follow this same approach. Pan et al. (2025) proposed a compact OCT classifier achieving 98.50% accuracy on the OCT-C8 dataset. Still, changes in recall and F1-score across certain classes suggest non-uniform performance. This, in turn, highlights the challenge of maintaining balanced metrics even when using lightweight architectures. Thus, the requirement for development of models that can guarantee accuracy as well as consistency in performance arises.

In this work, a YOLOv11-based classification framework referred to as the YOLOv11+PSF+SE+Head framework enhanced with Squeeze-and-Excitation (SE) blocks, a Progressive Spatial Fusion (PSF) module, and a custom classification head is proposed. The architecture leverages the strong feature extraction capability of YOLOv11 while improving multi-scale representation and interpretability through attention-guided refinement and Grad-CAM visualization. The main focus is to find a trade-off between the prediction accuracy, computation cost, and clinical applicability.

On OCT-C8 dataset, the proposed method gives 98.00% accuracy, precision, recall, and F1-score, along with 99.70% specificity (Table 11). The stability of the performance on all metrics proves that class-wise learning is achieved without any bias toward dominant categories. The statistical study using two-way ANOVA and Tukey's HSD test shows that the proposed method provides statistically significant improvements compared to Laouarem et al. (2024) in terms of precision and F1-score. The model also maintains comparable accuracy, recall, and specificity. These findings support the reliability of the proposed method.

From a computational feasibility perspective, the model has 9.06 million parameters and its size is 34.74 MB, which is lower than that of the ConvNeXt-Tiny, ResNet-101, and Vision Transformer-based models. Although the computational cost of the proposed framework is 14.25 GFLOPs, which is higher than several lightweight CNN architectures such as MobileNetV2, this additional computational overhead is introduced by the PSF module and attention refinement components to enable richer multi-scale feature integration and improved representation of disease-specific retinal patterns. The average inference time of 5.84 ms per image indicates the potential of the proposed framework for real-time OCT image analysis applications.

The framework outperforms current OCT-C8 models (Table 11) with an accuracy rate of 98.00%, equivalent to transformer and CNN models but with fewer parameters involved. Although Pan et al. (2025) achieved a slightly higher accuracy of 98.50%, their model had lower recall and F1-score values for several disease categories, indicating less balanced performance. Meanwhile, the proposed method has quite high and balanced measures in all aspects consistently, which is very important in medical situations where false negatives result in missed diagnoses.

The majority of errors, according to misclassification analysis, happen between visually similar classes, like CNV vs. Drusen and DME vs. Normal. Because early-stage retinal abnormalities have overlapping structural patterns, these confusions make clinical sense. Crucially, balanced macro-averaged metrics and the lack of class-specific bias show that the model does not overfit to dominant categories.

Additionally, the cross-dataset evaluation on the independent Kermany OCT2017 Version 2 dataset further shows the generalization ability of the proposed model in the domain shift context. The results of the proposed method show that the learned representations are not specific to the OCT-C8 dataset but are also applicable under different imaging conditions.

Despite achieving strong performance on benchmark OCT datasets, several limitations should be acknowledged. The proposed framework was evaluated using publicly available datasets and does not include prospective clinical testing, multi-center validation, or scanner-specific evaluation. Additionally, the OCT-C8 dataset used for primary evaluation provides image-level labels without lesion-level annotations or patient-specific metadata, limiting detailed lesion localization and patient-level clinical analysis. Variations in OCT acquisition devices, imaging protocols, and patient populations may introduce domain shifts that require further investigation. Although cross-dataset evaluation provides an initial assessment of generalization capability, additional evaluation using diverse clinical datasets is necessary before considering translation into real-world ophthalmic workflows. Furthermore, while the proposed framework provides automated classification and Grad-CAM-based visualization of model decision regions, further studies incorporating expert ophthalmologist evaluation and prospective clinical studies may provide additional insights into the clinical applicability and reliability of the proposed framework. Future studies may explore uncertainty estimation and calibration strategies to improve reliability assessment in clinical decision-support scenarios.

In summary, the proposed YOLOv11+PSF+SE+Head framework provides a balanced solution for eight-class OCT classification, achieving strong accuracy, robustness, and efficiency in a unified model demonstrating potential for real-time OCT-based screening support applications.

## 8 Conclusion

A YOLOv11-based framework incorporating a Progressive Spatial Fusion (PSF) module, Squeeze-and-Excitation (SE) blocks, and a customized classification head has been developed for eight-class retinal OCT image classification. The proposed method combines multi-scale feature fusion and attention-based refinement to improve the identification of retinal diseases while maintaining a favorable balance between classification performance and computational requirements. On the OCT-C8 dataset, experiments achieved 98.00% accuracy, precision, recall, and F1-score, with 99.70% specificity. Two-way ANOVA followed by Tukey's HSD *post hoc* tests confirmed that the difference in performance was statistically significant. The Grad-CAM analysis showed that the model was attending to retinal areas linked to disease characteristics, which provides a visual insight into the features learned by the model. The model was further validated on an independent OCT dataset where the model was able to generalize to a different data distribution. The results show that the proposed framework can provide accurate OCT-based retinal disease classification with interpretability and good generalization ability. Future work will involve evaluation of the framework on multi-center datasets and OCT images obtained from different devices, as well as the incorporation of multimodal clinical information to further improve computer-aided retinal disease assessment.

## Data Availability

The original contributions presented in the study are included in the article/supplementary material, further inquiries can be directed to the corresponding author.

## A Appendix

### A.1 Diabetic macular edema

DME is caused by fluid leaks from weakened retinal blood vessels and build-ups in the macula. This condition is a complication of diabetic retinopathy. In this condition chronic high blood sugar damages the retinal vasculature, leading to increased intra-retinal pressure and this causes fluid leakage into the retina. Recent studies indicate that the management of DME and patient outcomes can be significantly improved through early detection using advanced OCT imaging techniques (Pessoa et al., 2024).

### A.2 Choroidal neovascularization

The CNV arises from the proliferation of abnormal blood vessels originating in the choroid, breaching Bruch's membrane and extending into the subretinal pigment epithelium or the sub-retinal space. These pathological vessels tend to leak, causing progressive vision loss. Advanced OCT angiography techniques have provided clinicians with a greater understanding of the morphology of CNV, which has enabled earlier diagnosis and thus more accurate treatment planning (Feng et al., 2023).

### A.3 Drusen

Drusen are pathological extracellular deposits that progressively collect beneath the retinal pigment epithelium and are commonly associated with retinal degeneration. They are typically categorized into soft drusen, which consist of loosely organized granular or amorphous material and are considered potential early signs of AMD, and hard drusen, which are smaller, well defined, and usually linked to low risk, age related changes. The size, quantity, and accumulation of drusen impact the likelihood of progression to either atrophic (dry) or exudative (wet) AMD. Recent OCT based studies have helped to provide a more thorough understanding of drusen morphology and their role in the development of AMD (Sakurada et al., 2023).

### A.4 Macular hole

A complete defect in the fovea, the central area of the retina, is referred to as a MH. This condition typically results from vitreomacular traction or posterior vitreous detachment. The main cause for the defect is linked to aging. OCT plays a crucial role in giving detailed information about the size and stage of macular holes and helps in therapeutic decision making. The condition often causes distortion or blurring of central vision. Early detection through OCT facilitates prompt surgical intervention, such as vitrectomy, which has high success rates in restoring visual activity (Valentim et al., 2024).

### A.5 Diabetic Retinopathy

DR is a key microvascular complication of diabetes mellitus resulting from the slow breakdown of the retinal capillary system. OCT findings associated with DR includes intra retinal cystoid spaces, retinal thickening, hyperreflective foci, and the accumulation of exudate. Neovascularization and vitreous hemorrhage are potential complications of the progression of the disease. OCT and OCTA are important tools for disease staging and monitoring. Treatments include anti-VEGF therapy and laser treatment. Recent advances in deep learning now enable more accurate automated detection of hyperreflective foci in OCT images, improving both diagnostic precision and follow up assessments (Li et al., 2025).

### A.6 Central serous retinopathy

CSR is caused by the fluid accumulation beneath the neurosensory retina. This is due to the fluid originating from the choroid leaks through the RPE defects. Stress and corticosteroid use are some of the precise causes of CSR. OCT provides essential structural details, helping distinguish CSR from other retinal conditions like CNV. Recent advancements in AI have enhanced CSR detection and monitoring by enabling automated image analysis, which improves diagnostic accuracy (Zhang et al., 2025).

### A.7 Age-related macular degeneration

AMD is a main cause of vision loss in older adults. It consists of two main types which are called as dry and wet forms. The dry form is characterized by slow drusen accumulation and progressive atrophy of the retinal pigment epithelium leading to the gradual central vision loss. The wet form is defined by pigment epithelial detachment, the build-up of intraretinal or sub-retinal fluid, and choroidal neovascular membranes. This often causes a more rapid and severe decline in vision. OCT aids in diagnosing and monitoring AMD. It also helps in planning treatments, particularly for anti-VEGF therapies (Zang et al., 2023).

### A.8 Normal or healthy retina

The healthy retina serves as the primary reference for detecting unusual changes in the retinal imaging. High resolution OCT scans provide a clear view of retinal layers which makes it easier to identify structural abnormalities properly. Figure 1 illustrates a normal retinal OCT B scan with all major layers clearly annotated, offering a vital baseline for comparison. It is critical to create a normal retinal database using such imaging for proper diagnosis and disease monitoring (Jammal et al., 2022).
